# A Clustering Method with Historical Data to Support Large-Scale Consensus-Reaching Process in Group Decision-Making

**DOI:** 10.1007/s44196-022-00072-x

**Published:** 2022-03-19

**Authors:** Kai Xiong, Yucheng Dong, Sihai Zhao

**Affiliations:** 1 Center for Network Big Data and Decision-Making, Business School, Sichuan University, Chengdu, 610065 China; 2 School of Management, Shenzhen Institute of Information Technology, Shenzhen, 518172 China

**Keywords:** Large-scale group decision-making, Consensus-reaching process, K-means clustering, Historical data

## Abstract

With the rapid development of information technology and social network, the large-scale group decision-making (LSGDM) has become more and more popular due to the fact that large numbers of stakeholders are involved in different decision problems. To support the large-scale consensus-reaching process (LCRP), this paper proposes a LCRP framework based on a clustering method with the historical preference data of all decision makers (DMs). There are three parts in the proposed framework: the clustering process, the consensus process and the selection process. In the clustering process, we make use of an extended k-means clustering technique to divide the DMs into several clusters based on their historical preferences data. Next, the consensus process consists of the consensus measure and the feedback adjustment. The consensus measure aims to calculate the consensus level among DMs based on the obtained clusters. If the consensus level fails to reach the pre-defined consensus threshold, it is necessary to make the feedback adjustment to modify DMs' preferences. At last, the selection process is carried out to obtain a collective ranking of all alternatives. An illustrative example and detailed simulation experiments are demonstrated to show the validity of the proposed framework against the traditional LCRP models which just consider the preference information of DMs at only one stage for clustering.

## Introduction

Group decision-making (GDM) refers to a decision process where a group of decision makers (DMs) express their preferences regarding multiple alternatives and aim to obtain a ranking of the alternatives or select the best one(s) [1–3]. In GDM problems, the DMs usually express their opinions differently, while selecting a final solution with high consensus level among them is necessary. Therefore, the consensus-reaching process (CRP) was proposed and gradually became an effective tool to help DMs achieve a high consensus level, thus yielding a more acceptable solution [4–6]. Traditionally, the “hard” consensus in GDM considers only a full and unanimous agreement of DMs regarding the alternatives, which is sometimes time-consuming, difficult and unnecessary [7]. Therefore, the concept of “soft” consensus level [8, 9] was put forward and has been widely employed in different CRP models: (1) CRPs under different preference formats [6]; (2) CRPs with minimum adjustment or cost [10, 11]; (3) CRPs using consistency measure [12]; (4) CRPs considering the attitudes of DMs [13]; (5) CRPs under dynamic/Web contexts [14]; (6) CRPs based on trust relationship or DMs' weights [15, 16].

With the rapid development of information technology and social network, the increasing demands of organizations and the strengthening of the citizens' sense of democracy, more and more stakeholders are involved in different decision problems, which has led to the large-scale group decision-making (LSGDM) [17, 18]. Unlike traditional GDM problems which just involve a small number of DMs, LSGDM problems involve large numbers of DMs with various resources or information [19–21], and the number of DMs is usually no less than 20 in LSGDM events [22]. Generally speaking, decision outcome that may affect large numbers of groups or even the whole society is more appreciated if it is accepted by the masses with different backgrounds [23–25]. For example, the rescue plan about COVID-19 had great impact on the public, which might require the participation of large numbers of DMs from different fields. To date, the LSGDM has become a popular topic in decision analysis [26, 27], and many decision models were proposed to support the large-scale consensus-reaching process (LCRP). (1) LCRP addressing non-cooperative behaviors of DMs. For instance, Quesada et al. [27] proposed an expert weighting approach for the LCRP by incorporating the use of uninorm aggregation operators to manage experts' behaviors. Palomares et al. [28] incorporated a fuzzy clustering-based approach to detect and manage individual and subgroup non-cooperative behaviors in the LCRP. Furthermore, Gao and Zhang [29] proposed a consensus model to manage non-cooperative behaviors for personalized individual semantics-based social network GDM problems. (2) LCRP with individuals' different semantics or different formats of linguistic information. Li et al. [17] proposed a linguistic LCRP model based on personalized individual semantics, which took the individuals' different semantics into consideration. Zhang and Li [30] proposed a model for consistency control and consensus-reaching in linguistic GDM considering personalized individual semantics of DMs. Zhang et al. [31] proposed a linguistic distribution-based approach to deal with multiattribute LSGDM problems with multigranular unbalanced hesitant fuzzy linguistic information. (3) LCRP based on social network analysis. Liao et al. [32] proposed a LSGDM model based on social network analysis with probabilistic linguistic information. Ren et al. [33] developed a consensus model to manage minority opinions for LSGDM with social network analysis for micro-grid planning. There are also some other topics about LCRP. For instance, Wu and Xu [20] proposed a LCRP framework in which the fuzzy preference relations were used and the clusters were variable. Labella et al. [34] made a comparative study of different LCRP models by analyzing their different performances and sought out the main challenges of these models.

Although large numbers of LCRP models were proposed in the various studies, there are still some challenges which need to be pointed out.

1. To our knowledge, the clustering is a widely used tool in LCRP models, and the obtained clusters are usually determined by the preference information of DMs. The existing studies just consider the preference information of DMs at only one stage for clustering. In fact, the DMs' preference information is changing in all decision rounds, thus forming the historical preference information. By comparison, the historical preference information is more comprehensive, and may better guide the clustering process. For example, some DMs may tend to make their preferences more similar to other DMs' preferences when a large gap exists between the DM's own preference and other preferences. Then, the preferences of these DMs are getting closer and closer to the average level during the LCRP. From this point, the preference information of DMs at only one stage fails to fully reflect the change of DMs' preferences. Hence, the historical preference information of DMs in all decision rounds needs to be taken into consideration.
2. With the clustering method, the DMs are separated into a finite number of subgroups based on their preferences [35–37]. In most existing LCRP models, the clusters remain unchanged throughout the whole decision process [38, 39]. However, DMs' preferences are adjusted during the LCRP, so the clusters need to be variable in different decision stages. To our knowledge, only in few studies the clusters are variable, while their clusters are obtained based on the preference information of DMs at only one stage, rather than the historical preference information in all decision rounds. Therefore, it is important to propose a LCRP model where the clusters are variable in different decision stages through taking all the historical preference information of DMs into consideration.

To overcome the above challenges and shortcomings, this paper aims to propose a novel LCRP framework based on the clustering method using the historical preferences (CMHP) data of DMs. This framework is called CMHP-LCRP framework for simplicity. In the CMHP-LCRP framework, the historical preferences information of DMs in all decision rounds is used for clustering and the clusters are allowed to be variable accordingly in different decision stages. There are three parts in the proposed framework: the clustering process, the consensus process and the selection process. First, we divide the DMs into several clusters based on their historical preferences data. Next, it comes to the consensus process, which consists of the consensus measure and the feedback adjustment. The consensus level among DMs is measured based on the clustering results. If the consensus level fails to reach the pre-defined consensus threshold, the feedback adjustment strategy is employed to increase the consensus level among DMs. Otherwise, once the pre-defined consensus threshold is reached, the selection process is carried out to obtain a collective ranking of all alternatives. Finally, detailed simulation experiments and comparison analysis are demonstrated to show the validity of the CMHP-LCRP framework against the traditional LCRP models which just consider the preference information of DMs at only one stage for clustering.

This paper is organized as follows. Section 2 introduces some basic knowledge of GDM and the clustering technique. Then, the CMHP-LCRP framework is introduced in Sect. 3. Section 4 presents the clustering method, the consensus process and shows an illustrative example. Following this, in Sect. 5 the detailed simulation experiments and comparison analysis are demonstrated to show the validity of the CMHP-LCRP framework. Finally, concluding remarks and future studies are put forward in Sect. 6.

## Preliminaries

In this section, we introduce the basic knowledge of GDM problem and *k*-means clustering technique, which can lay the foundation for the rest of this study.

### GDM

We introduce the basic knowledge of GDM problem from the perspectives of preference relations, consensus process and selection process.

1. GDM with preference relations

In a GDM problem, there exist two basic elements: a finite set of DMs $E=\left\{{e}_{1},e,\dots ,{e}_{m}\right\}\left(m\ge 2\right)$, and a finite set of alternatives $X=\left\{{x}_{1},{x}_{2},\dots ,{x}_{n}\right\}\left(n\ge 2\right)$. Each DM expresses the evaluation over these alternatives using a kind of preference structure. On the whole, there are a lot of preference representation formats, such as utility functions [40], preference orderings [41], multiplicative preference relations [42], additive preference relations [43–45] and linguistic preference relations [46]. Additive preference relations format proves to be an effective and advantageous tool to represent the preferences of DMs and thus is widely used in GDM problems [9, 47–49]. In this paper, it is assumed that DMs use the additive preference relations to express their opinions.

#### Definition 1

Additive preference relations. Let ${B}^{k}={[{b}_{ij}^{k}]}_{n\times n}$ be an additive preference relation provided by DM ${e}_{k}\left(k=\mathrm{1,2},\dots ,m\right)$, where ${b}_{ij}^{k}$ denotes the degree of preference for alternative ${x}_{i}$ over alternative ${x}_{j}$ for DM ${e}_{k}$. Notably, ${b}_{ij}^{k}>0.5$ means ${x}_{i}$ is preferred to ${x}_{j}$, ${b}_{ij}^{k}<0.5$ means ${x}_{j}$ is preferred to ${x}_{i}$, and ${b}_{ij}^{k}=0.5$ means there is no difference between ${x}_{i}$ and ${x}_{j}$. The additive preference relations have the additive reciprocity property, ${b}_{ij}^{k}+{b}_{ji}^{k}=1,\forall i,j\in \left\{\mathrm{1,2},\dots ,n\right\}.$

1 $${B}^{k}=\left(\begin{array}{lll}{b}_{11}^{k}& \cdots & {b}_{1n}^{k}\\ \vdots & \ddots & \vdots \\ {b}_{n1}^{k}& \cdots & {b}_{nn}^{k}\end{array}\right).$$

Notably, the evaluation ${b}_{ii}^{k}$ $\left(i=\mathrm{1,2},\dots ,n\right)$, situated in the diagonal of the matrix, means the alternative ${x}_{i}$ is evaluated with itself, so the values in such diagonal are all 0.5.

2. Consensus process

In the existing literature, two processes are frequently used to solve GDM problems: consensus process and selection process.

The consensus process aims to raise the mutual agreement among DMs. There are different ways to interpret the consensus, such as the total agreement or some more flexible approaches. The consensus with a total agreement means the solution to the GDM problem is satisfactory for all DMs. In this situation, the cost might be unacceptable or the goal is sometimes hard to achieve in real life, which led to the development of soft consensus. In some studies, the flexible notion of consensus was proposed to soften the full and unanimous agreement [6, 50]. In this paper, a soft consensus-based approach is applied in LSGDM problems to obtain a satisfactory solution.

It is a dynamic and iterative discussion process to reach a certain degree of consensus in the CRP [8]. In general, there are two phases to achieve a consensus, which are described below. The first phase is to measure the consensus level that can reflect the degree of agreement among DMs based on their preferences. The second phase is feedback adjustment, which can provide guidance for DMs to modify their preferences and thus improve the degree of consensus among DMs. If the calculated consensus level does not reach the consensus threshold or it does not come to the maximum number of rounds allowed, the preferences resulting in disagreement are further recognized and the corresponding guidance for adjusting the DMs' preferences is provided so as to increase the consensus level in the next round.

3. Selection process

The selection process aims to obtain a ranking of the alternatives or select the best alternative(s). The selection process usually consists of two phases: the aggregation phase and the exploitation phase.

The aggregation phase aims to derive a collective preference through aggregating all the individuals' preference relations. Let ${P}^{c}={\left[{p}_{ij}^{c}\right]}_{n\times n}$ be the collective preference relation, where ${p}_{ij}^{c}$ denotes the group's preference degree of alternative ${x}_{i}$ over ${x}_{j}$. In this paper, the widely used weighted average (WA) operator [51, 52] is employed to carry out the aggregation operation.

2 $${p}_{ij}^{c}=WA\left({b}_{ij}^{1},{b}_{ij}^{2},\dots ,{b}_{ij}^{m}\right)=\sum \limits _{k=1}^{m}{w}_{k}{b}_{ij,}^{k}$$

where ${w}_{k}$ denotes the weight of DM ${e}_{k}\in E$ and ${\sum }_{k=1}^{m}{w}_{k}=1$, $0<{w}_{k}<1 \left(k=\mathrm{1,2},\dots ,m\right).$

The exploitation phase aims to obtain a ranking of all alternatives, which is implemented by deriving the collective preference over alternatives $X$ from the collective preference relation ${P}^{\mathrm{c}}={\left[{p}_{ij}^{\mathrm{c}}\right]}_{n\times n}$. Suppose ${pr}_{i}^{c}\ge 0$ means the collective preference over alternative ${x}_{i}$, and it is computed as follows.

3 $${pr}_{i}^{\mathrm{c}}=\frac{\sum_{j=1}^{n}{p}_{ij}^{\mathrm{c}}}{n}.$$

The larger the value of ${pr}_{i}^{\mathrm{c}}$, the better the alternative ${x}_{i}$. According to the Eq. (3), the ranking of all the alternatives can be obtained.

### *K*-means Clustering Technique

In the LCRP events, there are usually large numbers of DMs with diverse backgrounds and different preferences. For sake of analysis, these DMs are usually divided into several subgroups [20, 38, 53]. Clustering is a widely used tool contributing to data interpretation and analysis. With the clustering method, the data objects can be separated into a finite number of subgroups or clusters based on the similarity measure. In other words, the data objects in the same cluster share more similarities than those in different clusters. In the clustering process, each cluster is represented by a cluster center and the data belonging to such cluster show more similar characteristics. Many researches show that there are many clustering algorithms to compute the cluster centers and then determine the clustering results [54]. *K*-means clustering technique is one of the most popular clustering methods, and it also proves to be robust to determine the clustering result. Algorithm 1 shows the basic steps of the *k*-means clustering technique.

**Algorithm 1**. K-means clustering technique.

**Input**: The preference relations of all DMs ${B}^{k}={[{b}_{ij}^{k}]}_{n\times n} \left(k=\mathrm{1,2},\dots ,m\right)$ and the number of clusters $Q (Q\ge 2)$.

**Output**: Clusters $$\left\{{G}_{1},{G}_{2},\dots ,{G}_{Q}\right\}$$.

**Step 1:** Initialize $Q$ cluster centers ${C}^{h}={\left[{c}_{ij}^{h}\right]}_{n\times n}\left(h=\mathrm{1,2},\dots ,Q\right)$ from the preference relations of DMs ${B}^{k}={[{b}_{ij}^{k}]}_{n\times n}\left( k=\mathrm{1,2},\dots ,m\right)$ randomly as the initial central matrices.

**Step 2:** For the preference relation ${B}^{k}\left(k=\mathrm{1,2},\dots ,m\right)$, calculate the Euclidean distance between it and each cluster center ${C}^{h}\left(h=\mathrm{1,2},\dots ,Q\right)$, denoted as ${N}_{kh}$, where

4 $${N}_{kh}={\Vert {B}^{k}-{C}^{h}\Vert }_{2}=\sqrt{\sum \limits_{i=1}^{n-1} \sum \limits_{j=i+1}^{n}{\left({b}_{ij}^{k}-{c}_{ij}^{h}\right)}^{2}.}$$

${B}^{k}$ belongs to the corresponding cluster ${G}_{h}$ with the shortest distance ${N}_{kh}$.

**Step 3:** For each cluster ${G}_{h}$, update the center by averaging the preference relations ${B}^{k}$ assigned to it. Suppose the updated center is ${C}^{{h}^{^{\prime}}}={[{c}_{ij}^{{h}^{^{\prime}}}]}_{n\times n}$, then ${c}_{ij}^{{h}^{^{\prime}}}$=$\frac{1}{\left|{G}_{h}\right|}\sum_{{B}^{k}\in {G}_{h}}{b}_{ij}^{k}$, where $\left|{G}_{h}\right|$ is the number of preference relations assigned to ${G}_{h}$.

**Step 4:** Compute the Euclidean distance between the cluster ${C}^{h}$ and ${C}^{{h}^{^{\prime}}}$, which is denoted as $E$. The coefficient $\varepsilon >0$ is set as a convergence threshold, and one common criterion is $E\le \varepsilon$, where

5 $$E= \sum \limits_{h=1}^{Q}{\Vert {C}^{h}-{C}^{{h}^{^{\prime}}}\Vert }_{2}= \sum \limits_{h=1}^{Q}\sqrt{\sum \limits_{i=1}^{n-1} \sum \limits_{j=i+1}^{n}{\left({c}_{ij}^{h}-{c}_{ij}^{{h}^{^{\prime}}}\right)}^{2}.}$$

If the criterion is not met, iterate Step 2 and Step 3 until convergence, which means the cluster centers are invariable. Otherwise, output the clusters $\left\{{G}_{1},{G}_{2},\dots ,{G}_{Q}\right\}$.

## The Proposed CMHP-LCRP Framework

In this section, we present a flexible LCRP framework using the clustering method with the historical preferences data of DMs.

### Problem Description

As mentioned before, the clustering is a widely used tool to divide DMs into different clusters based on their preference relations in the LCRP. In the existing studies, the clustering process just considers the preference information of DMs at only one stage, which may fail to fully reflect the change of DMs' preferences. Therefore, it is of significance to take the historical preference information of DMs in all decision rounds into consideration and deal with it using proper theoretical models.

Recall that $X=\left\{{x}_{1},{x}_{2},\dots ,{x}_{n}\right\}\left(n\ge 2\right)$ is a finite set of alternatives, $E=\left\{{e}_{1},e,\dots ,{e}_{m}\right\}$ is a finite set of DMs,${B}^{k}={[{b}_{ij}^{k}]}_{n\times n}$ is the additive preference relation provided by DM ${e}_{k}$ on $X$, and $W={\left({w}_{1},{w}_{2},\dots ,{w}_{m}\right)}^{T}$ is a weight vector, where the weight of DM ${e}_{k}$ is denoted as ${w}_{k}$ and ${\sum }_{i=1}^{m}{w}_{k}=1$.

In the decision process, the DMs are adjusting their preferences in all decision rounds, thus forming the historical preference information, which can better reflect the change of DMs' preferences. Therefore, how to design a LCRP framework based on a clustering method with the historical preference data of DMs is of significance.

### The CMHP-LCRP Framework

**Fig. 1:**
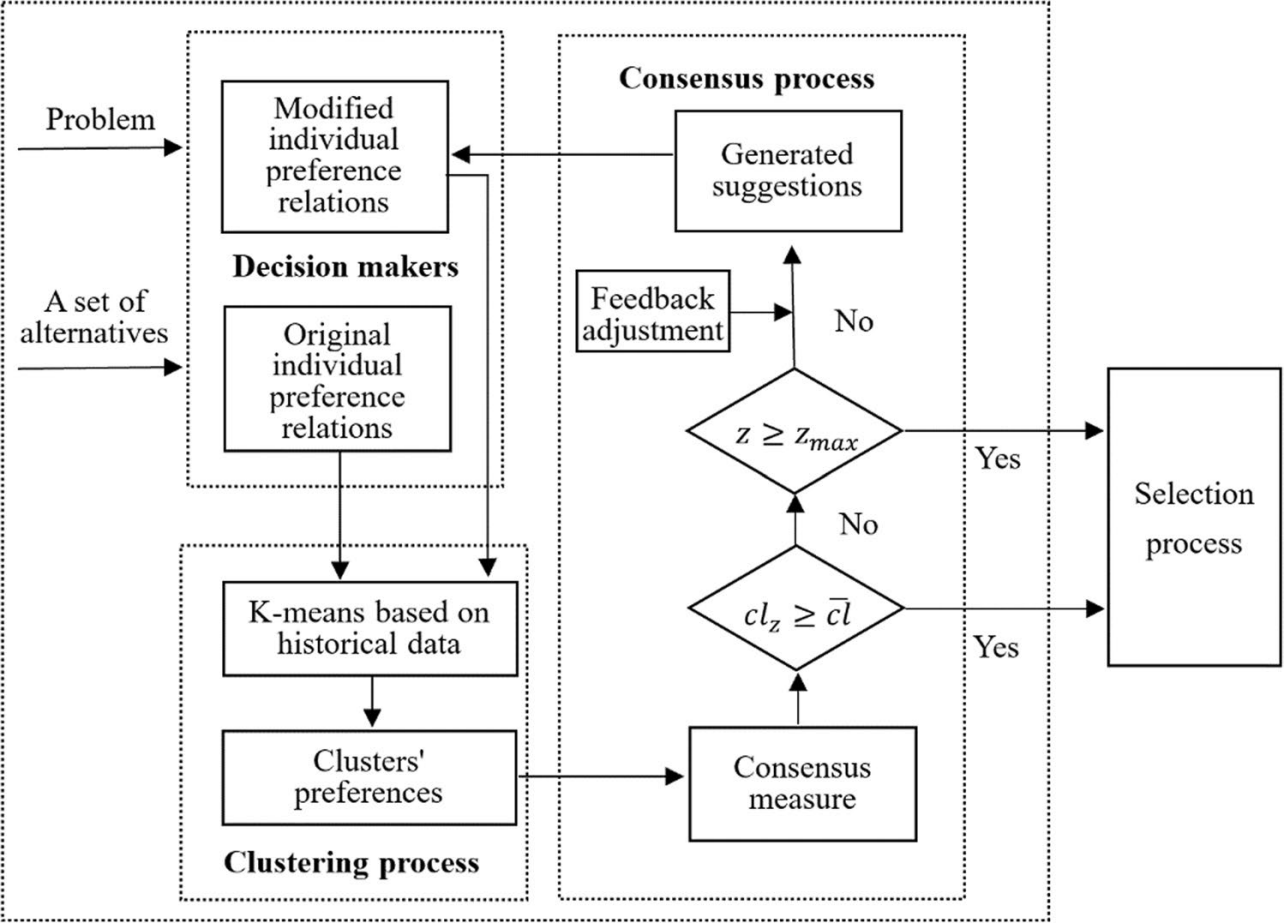
The CMHP-LCRP framework

Figure 1 shows the CMHP-LCRP framework in detail. On the whole, there are three main parts, namely, the clustering process, the consensus process and the selection process.

1. The clustering process

Clustering is a popular tool to divide DMs into several clusters and it has been widely used to deal with LSGDM problems. According to the obtained clusters, the analysis becomes much easier. There are a lot of clustering techniques, and k-means algorithm is one of the most popular techniques because of its high efficiency, simplicity and fast convergence [54]. In this paper, an extended *k*-means algorithm is used for clustering purpose. In previous literatures, the clusters were obtained just based on the preference information of DMs in the latest decision round, rather than the total historical preference data in all decision rounds. As the total historical preference information can reflect the characteristics of DMs' preferences more comprehensively, in this paper the historical preference data of DMs in all decision rounds are employed for clustering, which is quite different from previous studies.

2. The consensus process

For LCRP events, certain degree of consensus is essential. The consensus process aims to achieve the mutual agreement among DMs and it consists of the consensus measure and the feedback adjustment. The clustering process lays the foundation for the consensus measure. When the clusters are obtained, the consensus level among DMs can be calculated [55]. If the consensus level fails to reach the pre-defined consensus threshold or it does not come to the maximum number of rounds allowed, it is necessary to make the feedback adjustment strategy so as to achieve higher degree of consensus in the next round. Therefore, it is necessary to identify the DMs whose preference relations need to be adjusted. Based on this, some advice or suggestions are provided to modify the preference relations of DMs.

3. The selection process

If the pre-defined consensus threshold is reached or it comes to the maximum number of rounds allowed, the selection process is carried out to obtain a collective ranking of all alternatives.

## CMHP-LCRP Framework Analysis

In this section, we first present an extended k-means clustering method with the historical preference data. Next, the two main parts of LCRP, the consensus measure for obtained clusters and the corresponding feedback adjustment strategy are provided. Further, a detailed algorithm for the proposed CMHP-LCRP model to rank the alternatives is presented. At last, we use an example to illustrate the CMHP-LCRP model.

### The Extended K-means Clustering Method with the Historical Preference Data

1. The generation of historical preference data

*K*-means clustering technique proves to be robust to divide the DMs into several clusters and be widely used in the LSGDM problems. In this paper, an extended k-means clustering technique is used for clustering based on the DMs' historical preference data. Different from existing LCRP studies where clusters are usually obtained based on the preference information of DMs at only one stage, in this paper the historical preference data of DMs in all decision rounds are used for clustering. The detailed process of obtaining the historical preference data is described as follows.

Let $E=\left\{{e}_{1},e,\dots ,{e}_{m}\right\}$ and $X=\left\{{x}_{1},{x}_{2},\dots ,{x}_{n}\right\}\left(n\ge 2\right)$ be as before. The preference relation associated with DM ${e}_{k}$ on $X$ in round $z$ is denoted as ${B}^{k,z}$=${\left[{b}_{ij}^{k,z}\right]}_{n\times n}\left(k=\mathrm{1,2},\dots ,m\right)$. Next, we transform each preference relation ${B}^{k,z}$ into a vector that consists of its upper triangular elements, which is also denoted as ${B}^{k,z}$ for simplicity. Therefore,

6 $${B}^{k,z}=\left({b}_{12}^{k,z},{b}_{13}^{k,z},\dots ,{b}_{\left(n-1\right)n}^{k,z}\right).$$

Based on Eq. (6), the dimension of ${B}^{k,z}$ is $n(n-1)/2$. In order to further simplify ${B}^{k,z}$, suppose $y=n(n-1)/2$, and

7 $${B}^{k,z}=\left({b}_{1}^{k,z},{b}_{2}^{k,z},\dots ,{b}_{y}^{k,z}\right).$$

For the DM ${e}_{k}$, all the historical preference data $\left\{{B}^{k,z},{B}^{k,z-1},\dots ,{B}^{k,1}\right\}\left(k=\mathrm{1,2},\dots ,m\right)$ are used for clustering. Let all the historical preference of DM ${e}_{k}$ in round $z$ be ${H}^{k,z}\left(k=\mathrm{1,2},\dots ,m\right)$, which is presented as follows.

8 $${H}^{k,z}=\left({b}_{1}^{k,1},{b}_{2}^{k,1},\dots ,{b}_{y}^{k,1},{b}_{1}^{k,2},{b}_{2}^{k,2},\dots ,{b}_{y}^{k,2},\dots ,{b}_{1}^{k,z},{b}_{2}^{k,z},\dots ,{b}_{y}^{k,z}\right).$$

The dimension of ${H}^{k,z}$ is $zy$, and ${H}^{k,z}$ can be further transformed as follows.

9 $${H}^{k,z}=\left({b}_{1}^{k},{b}_{2}^{k},\dots ,{b}_{zy}^{k}\right).$$

Based on ${H}^{k,z}\left(k=\mathrm{1,2},\dots ,m\right)$, the DMs can be divided into different clusters, which lays the foundation for the LCRP analysis.

2. The extended *k*-means clustering technique based on the historical preference data

Since *k*-means clustering method is a widely used tool and it proves to be robust to determine the clustering result, therefore we use it to divide the DMs into several clusters according to the total historical preference data ${H}^{k,z}\left(k=\mathrm{1,2},\dots ,m\right)$. The procedures are as follows.

**Input**: All the historical preference relations ${H}^{k,z}=\left({b}_{1}^{k},{b}_{2}^{k},\dots ,{b}_{zy}^{k}\right)\left(k=\mathrm{1,2},\dots ,m\right)$ and the number of clusters $Q (Q\ge 2)$

**Output**: Clusters $$\left\{{G}_{1},{G}_{2},\dots ,{G}_{Q}\right\}.$$

**Step 1**: Initialize $Q$ cluster centers ${C}^{h}=\left({c}_{1}^{h},{c}_{2}^{h},\dots ,{c}_{zy}^{h}\right)\left(h=\mathrm{1,2},\dots ,Q\right)$ from all the historical preference relations ${H}^{k,z}=\left({b}_{1}^{k},{b}_{2}^{k},\dots ,{b}_{zy}^{k}\right)\left(k=\mathrm{1,2},\dots ,m\right)$ randomly as the initial central vectors.

**Step 2:** For the historical preference relation ${H}^{k,z}\left(k=\mathrm{1,2},\dots ,m\right)$, calculate the Euclidean distance between it and each cluster center ${C}^{h}\left(h=\mathrm{1,2},\dots ,Q\right)$, denoted as ${N}_{kh}$, where

10 $${N}_{kh}={\Vert {H}^{k,z}-{C}^{h}\Vert }_{2}=\sqrt{\sum \limits_{i=1}^{zy}{\left({b}_{i}^{k}-{c}_{i}^{h}\right)}^{2}}.$$

${H}^{k,z}$ belongs to the corresponding cluster ${G}_{h}$ with the shortest distance ${N}_{kh}$.

**Step 3:** For each cluster ${G}_{h}$, update the center by averaging all the historical preference relations ${H}^{k,z}$ assigned to it. Suppose the updated center is ${C}^{{h}^{^{\prime}}}=\left({c}_{1}^{{h}^{^{\prime}}},{c}_{2}^{{h}^{^{\prime}}},\dots ,{c}_{zy}^{{h}^{^{\prime}}}\right)$, then ${c}_{i}^{{h}^{^{\prime}}}$=$\frac{1}{\left|{G}_{h}\right|}\sum_{{H}^{k,z} \in {G}_{h}}{b}_{i}^{k}$, where $\left|{G}_{h}\right|$ is the number of historical preference relations assigned to ${G}_{h}$.

**Step 4:** Compute the Euclidean distance between the cluster ${C}^{h}$ and ${C}^{{h}^{^{\prime}}}$, which is denoted as $E$. The coefficient $\varepsilon >0$ is set as a convergence threshold, and one common criterion is $E\le \varepsilon$, where.

11 $$E=\sum\limits_{h=1}^{Q}{\Vert {C}^{h}-{C}^{{h}^{\mathrm{^{\prime}}}}\Vert }_{2}= \sum \limits_{h=1}^{Q}\sqrt{\sum \limits_{j=1}^{zy}{\left({c}_{j}^{h}-{c}_{j}^{{h}^{\mathrm{^{\prime}}}}\right)}^{2}}.$$

If the criterion is not met, iterate Step 2 and Step 3 until convergence. Otherwise, output the clusters $\left\{{G}_{1},{G}_{2},\dots ,{G}_{Q}\right\}$.

### The LCRP for the Proposed Framework

To achieve a certain degree of consensus in the CMHP-LCRP framework, there are usually two essential processes: consensus measure for clusters and local feedback adjustment strategy. At last, a detailed algorithm for the proposed CMHP-LCRP model to obtain the ranking of alternatives is presented.

#### Consensus Measure for Clusters

The consensus measure aims to calculate the degree of agreement among all DMs based on the obtained clusters. The steps to measure the consensus level of DMs are as follows.

1. Weight measure for clusters

Using the extended k-means clustering technique, the DMs are divided into several clusters, which are denoted as $\left\{{G}_{1},{G}_{2},\dots ,{G}_{Q}\right\}$. Let the number of DMs in cluster ${G}_{l}$ be ${m}_{l}$, ${m}_{l}>0$ and ${\sum }_{l=1}^{Q}{m}_{l}=m$. The DMs in cluster ${G}_{l}$ are denoted as $\left\{{e}_{1}^{{G}_{l}},{e}_{2}^{{G}_{l}},\dots ,{e}_{{m}_{l}}^{{G}_{l}}\right\}$. Suppose the weight for DM ${e}_{k}^{{G}_{l}}$ is ${w}_{k}^{{G}_{l}}$, and the weight of cluster ${G}_{l}$ can be calculated by adding all the weights of DMs in such cluster together, which is shown as follows.

12 $${w}^{{G}_{l}}={w}_{1}^{{G}_{l}}+{w}_{2}^{{G}_{l}}+\dots +{w}_{{m}_{l}}^{{G}_{l}}.$$

If the weights of all DMs are the same, then it follows that

13 $${w}^{{G}_{l}}= \sum \limits_{t=1}^{{m}_{l}}{w}_{t}^{{G}_{l}}=\frac{{m}_{l}}{m}.$$

Next, normalize the weight of each DM in cluster ${G}_{l}$. The normalized weight of ${e}_{k}^{{G}_{l}}$, denoted as ${u}_{k}^{{G}_{l}}$, is as follows

14 $${u}_{k}^{{G}_{l}}=\frac{{w}_{k}^{{G}_{l}}}{{\sum }_{t=1}^{{m}_{l}}{w}_{t}^{{G}_{l}}}=\frac{{w}_{k}^{{G}_{l}}}{{w}^{{G}_{l}}}.$$

2. Preference measure for clusters

The preference relation for cluster ${G}_{l}(l=\mathrm{1,2},\dots ,Q)$ is denoted as ${B}^{{G}_{l}}={\left[{b}_{ij}^{{G}_{l}}\right]}_{n\times n}(l=\mathrm{1,2},\dots ,Q)$, and it is a combination of the preferences of DMs in cluster ${G}_{l}$. Suppose the number of DMs in cluster ${G}_{l}$ is ${m}_{l}$, the preference relations in cluster ${G}_{l}$ for the pair of alternatives $\left({x}_{i},{x}_{j}\right)$ are denoted as $\left\{{b}_{ij}^{1,{G}_{l}},{b}_{ij}^{2,{G}_{l}},\dots ,{b}_{ij}^{{m}_{l},{G}_{l}}\right\}$, and the collective preference relation for cluster ${G}_{l}$ over the pair of alternatives $\left({x}_{i},{x}_{j}\right)$ can be presented as follows.

15 $${b}_{ij}^{{G}_{l}}=\sum_{k=1}^{{m}_{l}}{u}_{k}^{{G}_{l}}{b}_{ij}^{k,{G}_{l}}.$$

3. Similarity measure among clusters

For each pair of clusters ${G}_{s},{G}_{t}(s<t,\forall s,t\in \left\{\mathrm{1,2},\dots ,Q\right\})$, the similarity matrix ${\text{SM}}^{st}={\left({sm}_{ij}^{st}\right)}_{n\times n}$ is denoted as follows.

16 $${\text{SM}}^{st}=\left(\begin{array}{lll}-& \dots & {sm}_{1n}^{st}\\ \vdots & \ddots & \vdots \\ {sm}_{n1}^{st}& \dots & -\end{array}\right),$$

where ${sm}_{ij}^{st}$ measures the similarity degree between cluster ${G}_{s}$ and cluster ${G}_{t}$ over the pair of alternatives $\left({x}_{i},{x}_{j}\right)\left(i,j=\mathrm{1,2},\dots ,n\right)$, and the formula is represented as follows.

17 $${sm}_{ij}^{st}=1-\left|{b}_{ij}^{{G}_{s}}-{b}_{ij}^{{G}_{t}}\right|,$$

where $${sm}_{ij}^{st}={sm}_{ij}^{ts}$$.

4. Consensus measure

There are three different kinds of consensus degrees [23, 56], namely, consensus degree for each pair of alternatives, consensus degree for each alternative and consensus degree for the whole preference relations.

- Consensus degree for each pair of alternatives

For the pair of alternatives $({x}_{i},{x}_{j})$, the consensus degree is denoted as ${cm}_{ij}$, which is calculated by aggregating the similarity matrices associated with each pair of clusters $\left({G}_{s},{G}_{t}\right)$.

18 $${cm}_{ij}=\sum_{s=1}^{Q-1}\sum_{t=s+1}^{Q}{w}_{st}{sm}_{ij}^{st},$$

where ${w}_{st}$ is obtained by

19 $${w}_{st}=\frac{{w}^{{G}_{s}}{w}^{{G}_{t}}}{{\sum }_{s=1}^{Q-1}{\sum }_{t=s+1}^{Q}{w}^{{G}_{s}}{w}^{{G}_{t}}}.$$

${cm}_{ij}$ measures the consensus degree over the pair of alternatives $({x}_{i},{x}_{j})$. The larger the value of ${cm}_{ij}$, the higher the degree of agreement among DMs over the pair of alternatives $({x}_{i},{x}_{j})$. Such a measure helps to identify the pair of alternatives where the preferences need to be adjusted in the next round.

(b) Consensus degree for each alternative

For each alternative ${x}_{i}\left(i=\mathrm{1,2},\dots ,n\right)$, the consensus degree is denoted as ${ca}_{i}$, which is calculated by aggregating the consensus measures over each pair of alternatives.

20 $${ca}_{i}=\frac{{\sum }_{j=1,j\ne i}^{n}{cm}_{ij}}{n-1}.$$

${ca}_{i}$ measures the consensus degree for each alternative ${x}_{i}$. The larger the value of ${ca}_{i}$, the higher the degree of agreement among DMs over this alternative. Also, such a measure helps to identify the alternatives where the preferences need to be adjusted in the next round.

(iii) Consensus degree for the whole preference relations

The consensus degree for the whole preference relations is denoted $cl$, which is calculated by aggregating the consensus measures over each alternative.

21 $$cl=\frac{\sum_{i=1}^{n}{ca}_{i}}{n}.$$

$cl$ measures the global consensus level among all DMs. The larger the value of $cl$, the higher the degree of agreement among all DMs. Such a measure can determine whether to proceed to the next round by comparing such value with the consensus threshold.

#### Local Feedback Strategy

According to the consensus measure, we can calculate the global consensus level, which reflects the degree of agreement among all DMs. In general, there is a pre-defined consensus threshold. If the actual consensus level reaches the consensus threshold, which means the agreement among DMs is satisfactory, the consensus process ends and the selection process starts. Otherwise, DMs are urged to adjust the preferences in the next round so as to reach a higher consensus level. Overall, there are various models to adjust the preference of DMs. In this paper, a supervised mode, which requires the DMs to follow the following rules, is used.

The feedback adjustment strategy consists of two parts: the identification process and the adjustments process. The identification process aims to identify the preferences relations that need to be adjusted. Following this, the adjustments process gives the adjustment suggestions. The procedures are as follows.

1. The identification process.

The identification process consists of four identification rules, which help to precisely determine the alternatives, the pairs of alternatives, the clusters, and the DMs where the preferences need to be adjusted in the next round.

(a) Identification of alternatives. The first step is to identify the alternatives where the preferences need to be adjusted. Such alternatives are denoted as ${\text{CHX}}$, which satisfy the following rules.

22 $${\text{CHX}}=\left\{{x}_{i}|{ca}_{i}<cl,i=\mathrm{1,2},\dots ,n\right\}.$$

According to Eq. (20), ${ca}_{i}$ represents the consensus degree over the alternative ${x}_{i}\left(i=\mathrm{1,2},\dots ,n\right)$. If the value of ${ca}_{i}$ is smaller than the global consensus level, the preferences over the alternative ${x}_{i}$ are supposed to be adjusted in the next round so as to speed up the LCRP.

(b) Identification of the pairs of alternatives (positions). Based on the Eq. (22), the next step is to identify the pair of alternatives where the preferences need to be adjusted. Such pairs of alternatives are denoted as ${\text{CHP}}$, which satisfy the following rules.

23 $${\text{CHP}}=\left\{\left({x}_{i},{x}_{j}\right)|{x}_{i}\in {\text{CHX}} \; \mathrm{ and } \; {cm}_{ij}<cl,i<j\right\}.$$

According to Eq. (18), ${cm}_{ij}$ represents the consensus degree over the pair of alternatives $({x}_{i},{x}_{j})$. If the value of ${cm}_{ij}$ is smaller than the global consensus level, the preferences over the pair of alternatives $({x}_{i},{x}_{j})$ are supposed to be adjusted in the next round so as to speed up the LCRP.

(c) Identification of the best and worst clusters for each identified position. Based on Eq. (23), the next step is to further identify the best cluster and worst cluster for each identified position, which are denoted as ${\text{CLT}}_{ij}^{+}$ and ${\text{CLT}}_{ij}^{-}$, respectively. The two clusters satisfy the following rules.

24 $${\text{CLT}}_{ij}^{+}=\left\{{G}_{{s}^{+}}({x}_{i},{x}_{j})|{s}^{+}={\text{arg}}\;\underset{s}{\mathrm{max}}\left\{\sum \limits_{t=1,t\ne s}^{Q}{sm}_{ij}^{st}\right\}\right\},$$

25 $${\text{CLT}}_{ij}^{-}=\left\{{G}_{{s}^{\_}}({x}_{i},{x}_{j})|{s}^{-}={\text{arg}}\;\underset{s}{\mathrm{min}}\left\{ \sum \limits_{t=1,t\ne s}^{Q}{sm}_{ij}^{st}\right\}\right\},$$

There are just one best cluster and one worst cluster. To accelerate the LCRP, the DMs in all the other clusters except the best cluster ${\text{CLT}}_{ij}^{+}$ are supposed to adjust the preferences in the next round. The other clusters are denoted as ${{\text{CLT}}_{ij}^{-}}^{^{\prime}}$, which satisfy the following rules.

26 $${{\text{CLT}}_{ij}^{-}}^{^{\prime}}=\left\{{G}_{{s}^{\_}}({x}_{i},{x}_{j})|\left\{{G}_{1},{G}_{2},\dots ,{G}_{Q}\right\}\backslash {\text{CLT}}_{ij}^{+}\right\}.$$

(d) Identification of DMs. According Eqs. (23) and (26), the pairs of alternatives and the clusters where the preferences need to be adjusted have been identified. The DMs in ${{CLU}_{ij}^{-}}^{^{\prime}}$ are denoted as ${G}_{{s}^{-}}\left({x}_{i},{x}_{j}\right)=\left\{{e}_{{s}_{1}},{e}_{{s}_{2}},\dots ,{e}_{{s}_{G({s}^{-})}}\right\},$ where $G\left({s}^{-}\right)=\#\left({G}_{{S}^{-}}({x}_{i},{x}_{j})\right)$ is the number of DMs in ${{\text{CLT}}_{ij}^{-}}^{^{\prime}}$. Note that the preference relation of DM ${e}_{{s}_{y}}$ is denoted as ${B}^{{s}_{y}}={\left[{b}_{ij}^{{s}_{y}}\right]}_{n\times n}$. Meanwhile, the DMs in ${\text{CLT}}_{ij}^{+}$ are denoted ${G}_{{s}^{+}}\left({x}_{i},{x}_{j}\right)=\left\{{e}_{{t}_{1}},{e}_{{t}_{2}},\dots ,{e}_{{t}_{G({s}^{+})}}\right\},$ where $G\left({s}^{+}\right)=\#\left({G}_{{S}^{+}}({x}_{i},{x}_{j})\right)$ is the number of DMs in ${\text{CLT}}_{ij}^{+}$. The whole preference for the best cluster over the pair of alternatives $({x}_{i},{x}_{j})$ is as follows.

27 $${b}_{ij}^{{G}_{{s}^{+}}}=\sum_{l=1}^{{m}_{s+}}{u}_{l}^{{G}_{{s}^{+}}}{b}_{ij}^{l,{G}_{{s}^{+}}}.$$

Based on Eq. (27), a new parameter $\beta$ is set and the DMs satisfying the following conditions need to adjust the preferences over the pair of alternatives $({x}_{i},{x}_{j})$ in the next round.

28 $${E}_{ij}^{-}=\left\{{e}_{{s}_{y}}|\left|{b}_{ij}^{{s}_{y}}-{b}_{ij}^{{G}_{{s}^{+}}}\right|>\beta ,{s}_{y}\in \left\{{s}_{1},{s}_{2},\dots ,{s}_{G\left({s}^{\_}\right)}\right\},i<j\right\}.$$

According to Eq. (28), the DMs in ${E}_{ij}^{-}$ need to adjust their preferences over the pair of alternatives $({x}_{i},{x}_{j})$. The parameter $\beta$ plays the role of identifying the DMs that need to adjust the preferences. The larger the value of $\beta$, the fewer the DMs that need to adjust their preferences.

2. The adjustments process

Based on Eq. (28), the DMs that need to adjust their preferences have been identified. The next step is to adjust the preferences of the identified DMs. The updated preference of identified DM ${e}_{{s}_{y}}$ over the pair of alternatives $({x}_{i},{x}_{j})$ is denoted as ${b}_{ij}^{{s}_{{y}^{^{\prime}}}}$, which satisfies the following two conditions.

29 $${RB}^{{s}_{{y}^{^{\prime}}}}\left({x}_{i},{x}_{j}\right)=\left\{{b}_{ij}^{{s}_{{y}^{^{\prime}}}}|\left|{b}_{ij}^{{s}_{{y}^{^{\prime}}}}-{b}_{ij}^{{G}_{{s}^{+}}}\right|\le \beta ,i<j\right\},$$

30 $${RB}^{{s}_{{y}^{^{\prime}}}}\left({x}_{i},{x}_{j}\right)=\left\{{b}_{ij}^{{s}_{{y}^{^{\prime}}}}|{b}_{ij}^{{s}_{{y}^{^{\prime}}}}\in \left[min({b}_{ij}^{{s}_{y}},{b}_{ij}^{{G}_{{s}^{+}}}),max({b}_{ij}^{{s}_{y}},{b}_{ij}^{{G}_{{s}^{+}}})\right],i<j\right\}.$$

According to Eqs. (29) and (30), for any identified $i\mathrm{ and }j ( i<j),$ the updated preference relations follow certain direction and these updated preference relations become closer to the preference of the best cluster. Correspondingly, according to the rule of additive preference relations, ${b}_{ji}^{{s}_{{y}^{^{\prime}}}}$ is computed as follows.

31 $${b}_{ji}^{{s}_{{y}^{^{\prime}}}}=1-{b}_{ij}^{{s}_{{y}^{^{\prime}}}}, i<j.$$

#### Algorithm for the Proposed CMHP-LCRP Model

Based on the above, the details of the proposed CMHP-LCRP model are described in Algorithm 2.

**Algorithm 2**. CMHP-LCRP model.

**Input**: The preference relations of all DMs ${B}^{k}={[{b}_{ij}^{k}]}_{n\times n} \left(k=\mathrm{1,2},\dots ,m\right)$, the weights of DMs$\left\{{w}_{1},{w}_{2},\dots ,{w}_{m}\right\}$, the number of clusters$Q$, the parameter to adjust preference $\beta$ and the consensus threshold$\overline{cl }$.

**Output**: The ranking of alternatives.

**Step 1**: Let $z=1$, ${B}^{k,z}={B}^{k}$ and ${w}_{k,z}={w}_{k}\left(k=\mathrm{1,2},\dots ,m\right)$. According to the WA operator, aggregate all DMs' preference relations $\left\{{B}^{1,z},{B}^{2,z},\dots ,{B}^{m,z}\right\}$ to obtain the collective preference relation ${P}^{c,z}={\left[{p}_{ij}^{c,z}\right]}_{n\times n}$ based on Eq. (2).

**Step 2**: Gather the individuals' preference relations in each round $\left\{{B}^{k,z},{B}^{k,z-1},\dots ,{B}^{k,1}\right\}\left(k=\mathrm{1,2},\dots ,m\right)$ and transform them into the historical preference relations ${H}^{k,z}\left(k=\mathrm{1,2},\dots ,m\right)$ according to Eqs. (8) and (9).

**Step 3**: Based on the historical preference relations ${H}^{k,z}$, use the k-means clustering method to classify DMs into different clusters $\left\{{G}_{1},{G}_{2},\dots ,{G}_{Q}\right\}.$

**Step 4**: Calculate the global consensus level ${cl}_{z}$. If ${cl}_{z}\ge \overline{cl }$, go to step 6; otherwise continue with the next step.

**Step 5**: Identify the alternatives, the pairs of alternatives, the clusters and the DMs where the preferences need to be adjusted using the identification rules described in Sect. 4.2.2. The identified DMs are advised to adjust their preferences based on Eqs. (29)–(31), and back to step 2.

**Step 6**: Derive the ranking of alternatives from the evaluation values ${pr}_{i}^{c}=\frac{{\sum }_{j=1}^{n}{p}_{ij}^{c,z}}{n}$ according to Eq. (3). Output the ranking of alternatives.

### Illustrative Example

To demonstrate the applicability of the CMHP-LCRP framework, we further present an illustrative example. In this example, a set of twenty DMs $E=\left\{{e}_{1},{e}_{2},\dots ,{e}_{20}\right\}$ is assumed and a set of five alternatives $X=\left\{{x}_{1},{x}_{2},{x}_{3},{x}_{4},{x}_{5}\right\}$ are involved. The weights of all DMs are supposed to be the same, i.e., ${w}_{k}=0.05 (k=\mathrm{1,2},\dots ,20)$. Let $Q=3$, $\beta =0.1$ and $\overline{cl }=0.85$. The preference relations ${B}^{k}={\left[{b}_{ij}^{k}\right]}_{5\times 5}$ $\left(k=\mathrm{1,2},\dots ,20\right)$ are listed below.

$${B}^{\mathrm{1,1}}=\left(\begin{array}{lll}0.5& 0.56& \begin{array}{lll}0.10& 0.65& 0.88\end{array}\\ 0.44& 0.5& \begin{array}{lll}0.65& 0.96& 0.86\end{array}\\ \begin{array}{c}0.90\\ 0.35\\ 0.12\end{array}& \begin{array}{c}0.35\\ 0.04\\ 0.14\end{array}& \begin{array}{lll}0.5& 0.33& 0.30\\ 0.67& 0.5& 0.12\\ 0.70& 0.88& 0.5\end{array}\end{array}\right), \quad {B}^{\mathrm{2,1}}=\left(\begin{array}{lll}0.5& 0.35& \begin{array}{lll}0.47& 0.28& 0.50\end{array}\\ 0.65& 0.5& \begin{array}{lll}0.06& 0.61& 0.88\end{array}\\ \begin{array}{c}0.53\\ 0.72\\ 0.50\end{array}& \begin{array}{c}0.94\\ 0.39\\ 0.12\end{array}& \begin{array}{lll}0.5& 0.03& 0.78\\ 0.97& 0.5& 0.50\\ 0.22& 0.50& 0.5\end{array}\end{array}\right),$$

$${B}^{\mathrm{3,1}}=\left(\begin{array}{lll}0.5& 0.22& \begin{array}{lll}0.82& 0.89& 0.09\end{array}\\ 0.78& 0.5& \begin{array}{lll}0.14& 0.96& 0.46\end{array}\\ \begin{array}{c}0.18\\ 0.11\\ 0.91\end{array}& \begin{array}{c}0.86\\ 0.04\\ 0.54\end{array}& \begin{array}{lll}0.5& 0.93& 0.01\\ 0.07& 0.5& 0.28\\ 0.99& 0.72& 0.5\end{array}\end{array}\right), \quad {B}^{\mathrm{4,1}}=\left(\begin{array}{lll}0.5& 0.69& \begin{array}{lll}0.05& 0.02& 0.49\end{array}\\ 0.31& 0.5& \begin{array}{lll}0.57& 0.79& 0.81\end{array}\\ \begin{array}{c}0.95\\ 0.98\\ 0.51\end{array}& \begin{array}{c}0.43\\ 0.21\\ 0.19\end{array}& \begin{array}{lll}0.5& 0.17& 0.49\\ 0.83& 0.5& 0.95\\ 0.51& 0.05& 0.5\end{array}\end{array}\right),$$

$${B}^{\mathrm{5,1}}=\left(\begin{array}{lll}0.5& 0.87& \begin{array}{lll}0.83& 1.00& 0.58\end{array}\\ 0.13& 0.5& \begin{array}{lll}0.33& 0.98& 0.88\end{array}\\ \begin{array}{c}0.17\\ 0\\ 0.42\end{array}& \begin{array}{c}0.67\\ 0.02\\ 0.12\end{array}& \begin{array}{lll}0.5& 0.51& 0.14\\ 0.49& 0.5& 1.00\\ 0.86& 0& 0.5\end{array}\end{array}\right), \quad {B}^{\mathrm{6,1}}=\left(\begin{array}{lll}0.5& 0.43& \begin{array}{lll}0.02& 0.15& 0.72\end{array}\\ 0.57& 0.5& \begin{array}{lll}0.01& 0.92& 0.75\end{array}\\ \begin{array}{c}0.98\\ 0.85\\ 0.28\end{array}& \begin{array}{c}0.99\\ 0.08\\ 0.25\end{array}& \begin{array}{lll}0.5& 0.20& 0.26\\ 0.80& 0.5& 0.41\\ 0.74& 0.59& 0.5\end{array}\end{array}\right),$$

$${B}^{\mathrm{7,1}}=\left(\begin{array}{lll}0.5& 0.22& \begin{array}{lll}0.31& 0.53& 0.74\end{array}\\ 0.78& 0.5& \begin{array}{lll}0.68& 0.09& 0.11\end{array}\\ \begin{array}{c}0.69\\ 0.47\\ 0.26\end{array}& \begin{array}{c}0.32\\ 0.91\\ 0.89\end{array}& \begin{array}{lll}0.5& 0.48& 0.37\\ 0.52& 0.5& 0.18\\ 0.63& 0.82& 0.5\end{array}\end{array}\right), \quad {B}^{\mathrm{8,1}}=\left(\begin{array}{lll}0.5& 0.62& \begin{array}{lll}0.19& 0.76& 0.12\end{array}\\ 0.38& 0.5& \begin{array}{lll}0.75& 0.75& 0.23\end{array}\\ \begin{array}{c}0.81\\ 0.24\\ 0.88\end{array}& \begin{array}{c}0.25\\ 0.25\\ 0.77\end{array}& \begin{array}{lll}0.5& 0.25& 0.02\\ 0.75& 0.5& 0.54\\ 0.98& 0.46& 0.50\end{array}\end{array}\right),$$

$${B}^{\mathrm{9,1}}=\left(\begin{array}{lll}0.5& 0.27& \begin{array}{lll}0.48& 0.21& 0.80\end{array}\\ 0.73& 0.5& \begin{array}{lll}0.20& 0.32& 0.05\end{array}\\ \begin{array}{c}0.52\\ 0.79\\ 0.20\end{array}& \begin{array}{c}0.80\\ 0.68\\ 0.95\end{array}& \begin{array}{lll}0.5& 0.39& 0.68\\ 0.61& 0.5& 0.38\\ 0.32& 0.62& 0.5\end{array}\end{array}\right), \quad {B}^{\mathrm{10,1}}=\left(\begin{array}{lll}0.5& 0.94& \begin{array}{lll}0.25& 0.58& 0.19\end{array}\\ 0.06& 0.5& \begin{array}{lll}0.01& 0.28& 0.59\end{array}\\ \begin{array}{c}0.75\\ 0.42\\ 0.81\end{array}& \begin{array}{c}0.99\\ 0.72\\ 0.41\end{array}& \begin{array}{lll}0.5& 0.93& 0.88\\ 0.07& 0.5& 0.86\\ 0.12& 0.14& 0.5\end{array}\end{array}\right),$$

$${B}^{\mathrm{11,1}}=\left(\begin{array}{lll}0.5& 0.71& \begin{array}{lll}0.73& 0.41& 0.52\end{array}\\ 0.29& 0.5& \begin{array}{lll}0.06& 0.38& 0.72\end{array}\\ \begin{array}{c}0.27\\ 0.59\\ 0.48\end{array}& \begin{array}{c}0.94\\ 0.62\\ 0.28\end{array}& \begin{array}{lll}0.5& 0.12& 0.39\\ 0.88& 0.5& 0.56\\ 0.61& 0.44& 0.5\end{array}\end{array}\right), \quad {B}^{\mathrm{12,1}}=\left(\begin{array}{lll}0.5& 0.95& \begin{array}{lll}0.26& 0.96& 0.05\end{array}\\ 0.05& 0.5& \begin{array}{lll}0.49& 0.76& 0.74\end{array}\\ \begin{array}{c}0.74\\ 0.04\\ 0.95\end{array}& \begin{array}{c}0.51\\ 0.24\\ 0.26\end{array}& \begin{array}{lll}0.5& 0.25& 0.89\\ 0.75& 0.5& 0.91\\ 0.11& 0.09& 0.5\end{array}\end{array}\right),$$

$${B}^{\mathrm{13,1}}=\left(\begin{array}{lll}0.5& 0.69& \begin{array}{lll}0.60& 0.12& 0.30\end{array}\\ 0.31& 0.5& \begin{array}{lll}0.71& 0.72& 0.99\end{array}\\ \begin{array}{c}0.40\\ 0.88\\ 0.70\end{array}& \begin{array}{c}0.29\\ 0.28\\ 0.01\end{array}& \begin{array}{lll}0.5& 0.71& 0.76\\ 0.29& 0.5& 0.44\\ 0.24& 0.56& 0.5\end{array}\end{array}\right), \quad {B}^{\mathrm{14,1}}=\left(\begin{array}{lll}0.5& 0.67& \begin{array}{lll}0.25& 0.36& 0.83\end{array}\\ 0.33& 0.5& \begin{array}{lll}0.75& 0.42& 0.08\end{array}\\ \begin{array}{c}0.75\\ 0.64\\ 0.17\end{array}& \begin{array}{c}0.25\\ 0.58\\ 0.92\end{array}& \begin{array}{lll}0.5& 0.69& 0.94\\ 0.31& 0.5& 0.11\\ 0.06& 0.89& 0.5\end{array}\end{array}\right),$$

$${B}^{\mathrm{15,1}}=\left(\begin{array}{lll}0.5& 0.80& \begin{array}{lll}0.78& 0.02& 0.41\end{array}\\ 0.20& 0.5& \begin{array}{lll}0.10& 0.81& 1.00\end{array}\\ \begin{array}{c}0.22\\ 0.98\\ 0.59\end{array}& \begin{array}{c}0.90\\ 0.19\\ 0\end{array}& \begin{array}{lll}0.5& 0.96& 0.40\\ 0.04& 0.5& 0.05\\ 0.60& 0.95& 0.5\end{array}\end{array}\right), \quad {B}^{\mathrm{16,1}}=\left(\begin{array}{lll}0.5& 0.70& \begin{array}{lll}0.20& 0.20& 0.22\end{array}\\ 0.30& 0.5& \begin{array}{lll}0.29& 0.01& 0.84\end{array}\\ \begin{array}{c}0.80\\ 0.80\\ 0.78\end{array}& \begin{array}{c}0.71\\ 0.99\\ 0.16\end{array}& \begin{array}{lll}0.5& 0.15& 0.19\\ 0.85& 0.5& 0.22\\ 0.81& 0.78& 0.5\end{array}\end{array}\right),$$

$${B}^{\mathrm{17,1}}=\left(\begin{array}{lll}0.5& 0.37& \begin{array}{lll}0.42& 0.25& 0.85\end{array}\\ 0.63& 0.5& \begin{array}{lll}0.15& 0.66& 0.72\end{array}\\ \begin{array}{c}0.58\\ 0.75\\ 0.15\end{array}& \begin{array}{c}0.85\\ 0.34\\ 0.28\end{array}& \begin{array}{lll}0.5& 0.46& 0.13\\ 0.54& 0.5& 0.04\\ 0.87& 0.96& 0.5\end{array}\end{array}\right), \quad {B}^{\mathrm{18,1}}=\left(\begin{array}{lll}0.5& 0.55& \begin{array}{lll}0.99& 0.53& 0.05\end{array}\\ 0.45& 0.5& \begin{array}{lll}0.57& 0.17& 0.18\end{array}\\ \begin{array}{c}0.01\\ 0.47\\ 0.95\end{array}& \begin{array}{c}0.43\\ 0.83\\ 0.82\end{array}& \begin{array}{lll}0.5& 0.26& 0.26\\ 0.74& 0.5& 0.15\\ 0.74& 0.85& 0.5\end{array}\end{array}\right),$$

$${B}^{\mathrm{19,1}}=\left(\begin{array}{lll}0.5& 0.68& \begin{array}{lll}0.89& 0.62& 0.67\end{array}\\ 0.32& 0.5& \begin{array}{lll}0.47& 0.48& 0.23\end{array}\\ \begin{array}{c}0.11\\ 0.38\\ 0.33\end{array}& \begin{array}{c}0.53\\ 0.52\\ 0.77\end{array}& \begin{array}{lll}0.5& 0.67& 0.43\\ 0.33& 0.5& 0.80\\ 0.57& 0.20& 0.5\end{array}\end{array}\right), \quad {B}^{\mathrm{20,1}}=\left(\begin{array}{lll}0.5& 0.43& \begin{array}{lll}0.59& 0.99& 0.30\end{array}\\ 0.57& 0.5& \begin{array}{lll}0.94& 0.64& 0.77\end{array}\\ \begin{array}{c}0.41\\ 0.01\\ 0.70\end{array}& \begin{array}{c}0.06\\ 0.36\\ 0.23\end{array}& \begin{array}{lll}0.5& 0.95& 0.62\\ 0.05& 0.5& 0.67\\ 0.38& 0.33& 0.5\end{array}\end{array}\right).$$

In the following, the proposed CMHP-LCRP framework is employed to show the LCRP.

### First Round

In this example, three clusters are set for analysis. Based on individuals' preference relations, use the k-means clustering technique to classify DMs into the following clusters.

$${G}_{1}=\left\{{B}^{\mathrm{1,1}}, \;{B}^{\mathrm{2,1}},\; {B}^{\mathrm{6,1}}, \; {B}^{\mathrm{7,1}}, \; {B}^{\mathrm{9,1}}, \; {B}^{\mathrm{11,1}}, \; {B}^{\mathrm{14,1}}, \; {B}^{\mathrm{16,1}}, \; {B}^{\mathrm{17,1}}\right\}$$

$${G}_{2}=\left\{{B}^{\mathrm{3,1}},{B}^{\mathrm{13,1}},{B}^{\mathrm{15,1}},{B}^{\mathrm{18,1}}\right\},$$

$${G}_{3}=\left\{{B}^{\mathrm{4,1}},{B}^{\mathrm{5,1}},{B}^{\mathrm{8,1}},{B}^{\mathrm{10,1}},{B}^{\mathrm{12,1}},{B}^{\mathrm{19,1}},{B}^{\mathrm{20,1}}\right\}.$$

Based on Eq. (15), we can obtain the collective preferences for each cluster.

$${B}^{{G}_{1}}=\left(\begin{array}{lll}0.5& 0.4756& \begin{array}{lll}0.3311& 0.3378& 0.6733\end{array}\\ 0.5244& 0.5& \begin{array}{lll}0.3167& 0.4856& 0.5567\end{array}\\ \begin{array}{c}0.6689\\ 0.6622\\ 0.3267\end{array}& \begin{array}{c}0.6833\\ 0.5144\\ 0.4433\end{array}& \begin{array}{lll}0.5& 0.3167& 0.4489\\ 0.6833& 0.5& 0.2800\\ 0.5511& 0.7200& 0.5\end{array}\end{array}\right),$$

$${B}^{{G}_{2}}=\left(\begin{array}{lll}0.5& 0.5650& \begin{array}{lll}0.7975& 0.3900& 0.2125\end{array}\\ 0.4350& 0.5& \begin{array}{lll}0.3800& 0.6650& 0.6575\end{array}\\ \begin{array}{c}0.2025\\ 0.6100\\ 0.7875\end{array}& \begin{array}{c}0.6200\\ 0.3350\\ 0.3425\end{array}& \begin{array}{lll}0.5& 0.7150& 0.3575\\ 0.2850& 0.5& 0.2300\\ 0.6425& 0.7700& 0.5\end{array}\end{array}\right),$$

$${B}^{{G}_{3}}=\left(\begin{array}{lll}0.5& 0.7400& \begin{array}{lll}0.4371& 0.7043& 0.3429\end{array}\\ 0.2600& 0.5& \begin{array}{lll}0.5086& 0.6686& 0.6071\end{array}\\ \begin{array}{c}0.5629\\ 0.2957\\ 0.6571\end{array}& \begin{array}{c}0.4914\\ 0.3314\\ 0.3929\end{array}& \begin{array}{lll}0.5& 0.5329& 0.4957\\ 0.4671& 0.5& 0.8186\\ 0.5043& 0.1814& 0.5\end{array}\end{array}\right).$$

Based on Eq. (17), the similarity matrices are obtained as follows.

$${\text{SM}}_{12}=\left(\begin{array}{lll}-& 0.9106& \begin{array}{lll}0.5336& 0.9478& 0.5392\end{array}\\ 0.9106& -& \begin{array}{lll}0.9367& 0.8206& 0.8992\end{array}\\ \begin{array}{c}0.5336\\ 0.9478\\ 0.5392\end{array}& \begin{array}{c}0.9367\\ 0.8206\\ 0.8992\end{array}& \begin{array}{lll}-& 0.6017& 0.9086\\ 0.6017& -& 0.9500\\ 0.9086& 0.9500& -\end{array}\end{array}\right),$$

$${\text{SM}}_{13}=\left(\begin{array}{lll}-& 0.7356& \begin{array}{lll}0.8940& 0.6335& 0.6695\end{array}\\ 0.7356& -& \begin{array}{lll}0.80801& 0.8170& 0.9495\end{array}\\ \begin{array}{c}0.8940\\ 0.6335\\ 0.6695\end{array}& \begin{array}{c}0.8081\\ 0.8170\\ 0.9495\end{array}& \begin{array}{lll}-& 0.7838& 0.9532\\ 0.7838& -& 0.4614\\ 0.9532& 0.4614& -\end{array}\end{array}\right),$$

$${\text{SM}}_{23}=\left(\begin{array}{lll}-& 0.8250& \begin{array}{lll}0.6396& 0.6857& 0.8696\end{array}\\ 0.8250& -& \begin{array}{lll}0.8714& 0.9964& 0.9496\end{array}\\ \begin{array}{c}0.6396\\ 0.6857\\ 0.8696\end{array}& \begin{array}{c}0.8714\\ 0.9964\\ 0.9496\end{array}& \begin{array}{lll}-& 0.8179& 0.8618\\ 0.8179& -& 0.4114\\ 0.8618& 0.4114& -\end{array}\end{array}\right).$$

From Eqs. (18)–(20), we can obtain the consensus degrees for each alternative.

$${ca}_{1}=0.7379, {ca}_{2}=0.8641, {ca}_{3}=0.8136, {ca}_{4}=0.7301, {ca}_{5}=0.7803.$$

Therefore, according to Eq. (21) the global consensus level is calculated.

$${cl}_{1}=0.7852.$$

As ${cl}_{1}<0.85$, the feedback adjustment strategy is provided. As shown above, the consensus degrees for each alternative as well as those for each pair of alternatives are obtained. According to Eq. (22), we conclude the preferences over the alternatives ${x}_{1}$ and ${x}_{4}$ are supposed to be adjusted. According to Eq. (23), we further conclude the preferences over these pairs of alternatives $\left({x}_{1},{x}_{3}\right)$, $\left({x}_{1},{x}_{4}\right)$, $\left({x}_{1},{x}_{5}\right)$ and $\left({x}_{4},{x}_{5}\right)$ need to be adjusted. Then, the next step is to identify the best and worst clusters for each identified position. In order to accelerate the LCRP, in this paper the DMs in all the other clusters except the best cluster are supposed to adjust the preferences. Based on Eqs. (24) and (26), the ideal cluster and all the other clusters are presented as follows.

$${\text{CLT}}_{13}^{+}=\left\{{G}_{3}\right\}, {\text{CLT}}_{13}^{-\prime}=\left\{{G}_{1},{G}_{2}\right\},$$

$${\text{CLT}}_{14}^{+}=\left\{{G}_{2}\right\}, {\text{CLT}}_{14}^{-\prime}=\left\{{G}_{1},{G}_{3}\right\},$$

$${\text{CLT}}_{15}^{+}=\left\{{G}_{3}\right\}, {\text{CLT}}_{15}^{- \prime}=\left\{{G}_{1},{G}_{2}\right\},$$

$${\text{CLT}}_{45}^{+}=\left\{{G}_{1}\right\}, {\text{CLT}}_{45}^{- \prime}=\left\{{G}_{2},{G}_{3}\right\}.$$

### Second Round

In the example $\beta =0.1$, from Eqs. (29)–(31) the DMs who need to adjust the preferences are identified, and the updated preferences for DMs are listed below.

$${B}^{\mathrm{1,2}}=\left(\begin{array}{lll}0.5& 0.56& \begin{array}{lll}0.36& 0.44& 0.40\end{array}\\ 0.44& 0.5& \begin{array}{lll}0.65& 0.96& 0.86\end{array}\\ \begin{array}{c}0.64\\ 0.56\\ 0.60\end{array}& \begin{array}{c}0.35\\ 0.04\\ 0.14\end{array}& \begin{array}{lll}0.5& 0.33& 0.30\\ 0.67& 0.5& 0.12\\ 0.70& 0.88& 0.5\end{array}\end{array}\right), \quad {B}^{\mathrm{2,2}}=\left(\begin{array}{lll}0.5& 0.35& \begin{array}{lll}0.47& 0.36& 0.38\end{array}\\ 0.65& 0.5& \begin{array}{lll}0.06& 0.61& 0.88\end{array}\\ \begin{array}{c}0.53\\ 0.64\\ 0.62\end{array}& \begin{array}{c}0.94\\ 0.39\\ 0.12\end{array}& \begin{array}{lll}0.5& 0.03& 0.78\\ 0.97& 0.5& 0.50\\ 0.22& 0.50& 0.5\end{array}\end{array}\right),$$

$${B}^{\mathrm{3,2}}=\left(\begin{array}{lll}0.5& 0.22& \begin{array}{lll}0.51& 0.89& 0.33\end{array}\\ 0.78& 0.5& \begin{array}{lll}0.14& 0.96& 0.46\end{array}\\ \begin{array}{c}0.49\\ 0.11\\ 0.67\end{array}& \begin{array}{c}0.86\\ 0.04\\ 0.54\end{array}& \begin{array}{lll}0.5& 0.93& 0.01\\ 0.07& 0.5& 0.28\\ 0.99& 0.72& 0.5\end{array}\end{array}\right), \quad {B}^{\mathrm{4,2}}=\left(\begin{array}{lll}0.5& 0.69& \begin{array}{lll}0.05& 0.32& 0.49\end{array}\\ 0.31& 0.5& \begin{array}{lll}0.57& 0.79& 0.81\end{array}\\ \begin{array}{c}0.95\\ 0.68\\ 0.51\end{array}& \begin{array}{c}0.43\\ 0.21\\ 0.19\end{array}& \begin{array}{lll}0.5& 0.17& 0.49\\ 0.83& 0.5& 0.33\\ 0.51& 0.67& 0.5\end{array}\end{array}\right),$$

$${B}^{\mathrm{5,2}}=\left(\begin{array}{lll}0.5& 0.87& \begin{array}{lll}0.83& 0.49& 0.58\end{array}\\ 0.13& 0.5& \begin{array}{lll}0.33& 0.98& 0.88\end{array}\\ \begin{array}{c}0.17\\ 0.51\\ 0.42\end{array}& \begin{array}{c}0.67\\ 0.02\\ 0.12\end{array}& \begin{array}{lll}0.5& 0.51& 0.14\\ 0.49& 0.5& 0.36\\ 0.86& 0.64& 0.5\end{array}\end{array}\right), \quad {B}^{\mathrm{6,2}}=\left(\begin{array}{lll}0.5& 0.43& \begin{array}{lll}0.40& 0.31& 0.42\end{array}\\ 0.57& 0.5& \begin{array}{lll}0.01& 0.92& 0.75\end{array}\\ \begin{array}{c}0.60\\ 0.69\\ 0.58\end{array}& \begin{array}{c}0.99\\ 0.08\\ 0.25\end{array}& \begin{array}{lll}0.5& 0.20& 0.26\\ 0.80& 0.5& 0.41\\ 0.74& 0.59& 0.5\end{array}\end{array}\right),$$

$${B}^{\mathrm{7,2}}=\left(\begin{array}{lll}0.5& 0.22& \begin{array}{lll}0.41& 0.47& 0.40\end{array}\\ 0.78& 0.5& \begin{array}{lll}0.68& 0.09& 0.11\end{array}\\ \begin{array}{c}0.59\\ 0.53\\ 0.60\end{array}& \begin{array}{c}0.32\\ 0.91\\ 0.89\end{array}& \begin{array}{lll}0.5& 0.48& 0.37\\ 0.52& 0.5& 0.18\\ 0.63& 0.82& 0.5\end{array}\end{array}\right), \quad {B}^{\mathrm{8,2}}=\left(\begin{array}{lll}0.5& 0.62& \begin{array}{lll}0.19& 0.46& 0.12\end{array}\\ 0.38& 0.5& \begin{array}{lll}0.75& 0.75& 0.23\end{array}\\ \begin{array}{c}0.81\\ 0.54\\ 0.88\end{array}& \begin{array}{c}0.25\\ 0.25\\ 0.77\end{array}& \begin{array}{lll}0.5& 0.25& 0.02\\ 0.75& 0.5& 0.33\\ 0.98& 0.67& 0.50\end{array}\end{array}\right),$$

$${B}^{\mathrm{9,2}}=\left(\begin{array}{lll}0.5& 0.27& \begin{array}{lll}0.48& 0.33& 0.42\end{array}\\ 0.73& 0.5& \begin{array}{lll}0.20& 0.32& 0.05\end{array}\\ \begin{array}{c}0.52\\ 0.67\\ 0.58\end{array}& \begin{array}{c}0.80\\ 0.68\\ 0.95\end{array}& \begin{array}{lll}0.5& 0.39& 0.68\\ 0.61& 0.5& 0.38\\ 0.32& 0.62& 0.5\end{array}\end{array}\right), \quad {B}^{\mathrm{10,2}}=\left(\begin{array}{lll}0.5& 0.94& \begin{array}{lll}0.25& 0.47& 0.19\end{array}\\ 0.06& 0.5& \begin{array}{lll}0.01& 0.28& 0.59\end{array}\\ \begin{array}{c}0.75\\ 0.53\\ 0.81\end{array}& \begin{array}{c}0.99\\ 0.72\\ 0.41\end{array}& \begin{array}{lll}0.5& 0.93& 0.88\\ 0.07& 0.5& 0.36\\ 0.12& 0.64& 0.5\end{array}\end{array}\right),$$

$${B}^{\mathrm{11,2}}=\left(\begin{array}{lll}0.5& 0.71& \begin{array}{lll}0.49& 0.41& 0.40\end{array}\\ 0.29& 0.5& \begin{array}{lll}0.06& 0.38& 0.72\end{array}\\ \begin{array}{c}0.51\\ 0.59\\ 0.60\end{array}& \begin{array}{c}0.94\\ 0.62\\ 0.28\end{array}& \begin{array}{lll}0.5& 0.12& 0.39\\ 0.88& 0.5& 0.56\\ 0.61& 0.44& 0.5\end{array}\end{array}\right), \quad {B}^{\mathrm{12,2}}=\left(\begin{array}{lll}0.5& 0.95& \begin{array}{lll}0.26& 0.44& 0.05\end{array}\\ 0.05& 0.5& \begin{array}{lll}0.49& 0.76& 0.74\end{array}\\ \begin{array}{c}0.74\\ 0.56\\ 0.95\end{array}& \begin{array}{c}0.51\\ 0.24\\ 0.26\end{array}& \begin{array}{lll}0.5& 0.25& 0.89\\ 0.75& 0.5& 0.31\\ 0.11& 0.69& 0.5\end{array}\end{array}\right),$$

$${B}^{\mathrm{13,2}}=\left(\begin{array}{lll}0.5& 0.69& \begin{array}{lll}0.46& 0.12& 0.30\end{array}\\ 0.31& 0.5& \begin{array}{lll}0.71& 0.72& 0.99\end{array}\\ \begin{array}{c}0.54\\ 0.88\\ 0.70\end{array}& \begin{array}{c}0.29\\ 0.28\\ 0.01\end{array}& \begin{array}{lll}0.5& 0.71& 0.76\\ 0.29& 0.5& 0.38\\ 0.24& 0.62& 0.5\end{array}\end{array}\right), \quad {B}^{\mathrm{14,2}}=\left(\begin{array}{lll}0.5& 0.67& \begin{array}{lll}0.35& 0.36& 0.42\end{array}\\ 0.33& 0.5& \begin{array}{lll}0.75& 0.42& 0.08\end{array}\\ \begin{array}{c}0.65\\ 0.64\\ 0.58\end{array}& \begin{array}{c}0.25\\ 0.58\\ 0.92\end{array}& \begin{array}{lll}0.5& 0.69& 0.94\\ 0.31& 0.5& 0.11\\ 0.06& 0.89& 0.5\end{array}\end{array}\right),$$

$${B}^{\mathrm{15,2}}=\left(\begin{array}{lll}0.5& 0.80& \begin{array}{lll}0.50& 0.02& 0.41\end{array}\\ 0.20& 0.5& \begin{array}{lll}0.10& 0.81& 1.00\end{array}\\ \begin{array}{c}0.50\\ 0.98\\ 0.59\end{array}& \begin{array}{c}0.90\\ 0.19\\ 0\end{array}& \begin{array}{lll}0.5& 0.96& 0.40\\ 0.04& 0.5& 0.25\\ 0.60& 0.75& 0.5\end{array}\end{array}\right), \quad {B}^{\mathrm{16,2}}=\left(\begin{array}{lll}0.5& 0.70& \begin{array}{lll}0.36& 0.30& 0.29\end{array}\\ 0.30& 0.5& \begin{array}{lll}0.29& 0.01& 0.84\end{array}\\ \begin{array}{c}0.64\\ 0.70\\ 0.71\end{array}& \begin{array}{c}0.71\\ 0.99\\ 0.16\end{array}& \begin{array}{lll}0.5& 0.15& 0.19\\ 0.85& 0.5& 0.22\\ 0.81& 0.78& 0.5\end{array}\end{array}\right),$$

$${B}^{\mathrm{17,2}}=\left(\begin{array}{lll}0.5& 0.37& \begin{array}{lll}0.42& 0.38& 0.44\end{array}\\ 0.63& 0.5& \begin{array}{lll}0.15& 0.66& 0.72\end{array}\\ \begin{array}{c}0.58\\ 0.62\\ 0.56\end{array}& \begin{array}{c}0.85\\ 0.34\\ 0.28\end{array}& \begin{array}{lll}0.5& 0.46& 0.13\\ 0.54& 0.5& 0.04\\ 0.87& 0.96& 0.5\end{array}\end{array}\right), \quad {B}^{\mathrm{18,2}}=\left(\begin{array}{lll}0.5& 0.55& \begin{array}{lll}0.52& 0.53& 0.26\end{array}\\ 0.45& 0.5& \begin{array}{lll}0.57& 0.17& 0.18\end{array}\\ \begin{array}{c}0.48\\ 0.47\\ 0.74\end{array}& \begin{array}{c}0.43\\ 0.83\\ 0.82\end{array}& \begin{array}{lll}0.5& 0.26& 0.26\\ 0.74& 0.5& 0.27\\ 0.74& 0.73& 0.5\end{array}\end{array}\right),$$

$${B}^{\mathrm{19,2}}=\left(\begin{array}{lll}0.5& 0.68& \begin{array}{lll}0.89& 0.49& 0.67\end{array}\\ 0.32& 0.5& \begin{array}{lll}0.47& 0.48& 0.23\end{array}\\ \begin{array}{c}0.11\\ 0.51\\ 0.33\end{array}& \begin{array}{c}0.53\\ 0.52\\ 0.77\end{array}& \begin{array}{lll}0.5& 0.67& 0.43\\ 0.33& 0.5& 0.36\\ 0.57& 0.64& 0.5\end{array}\end{array}\right), \quad {B}^{\mathrm{20,2}}=\left(\begin{array}{lll}0.5& 0.43& \begin{array}{lll}0.59& 0.40& 0.30\end{array}\\ 0.57& 0.5& \begin{array}{lll}0.94& 0.64& 0.77\end{array}\\ \begin{array}{c}0.41\\ 0.50\\ 0.70\end{array}& \begin{array}{c}0.06\\ 0.36\\ 0.23\end{array}& \begin{array}{lll}0.5& 0.95& 0.62\\ 0.05& 0.5& 0.36\\ 0.38& 0.64& 0.5\end{array}\end{array}\right).$$

Based on the historical preference relations, use the k-means clustering technique to classify DMs into the following clusters.

$${G}_{1}=\left\{{B}^{\mathrm{3,2}},{B}^{\mathrm{5,2}},{B}^{\mathrm{7,2}},{B}^{8,2},{B}^{\mathrm{9,2}},{B}^{\mathrm{10,2}},{B}^{\mathrm{12,2}},{B}^{\mathrm{14,2}},{B}^{\mathrm{18,2}},{B}^{\mathrm{19,2}},{B}^{\mathrm{20,2}}\right\},$$

$${G}_{2}=\left\{{B}^{\mathrm{1,2}},{B}^{\mathrm{2,2}},{B}^{\mathrm{4,2}},{B}^{\mathrm{6,2}},{B}^{\mathrm{11,2}},{B}^{\mathrm{16,2}},{B}^{\mathrm{17,2}}\right\},$$

$${G}_{3}=\left\{{B}^{\mathrm{13,2}},{B}^{\mathrm{15,2}}\right\}.$$

Based on Eq. (15), we can obtain the collective preferences for each cluster.

$${B}^{{G}_{1}}=\left(\begin{array}{lll}0.5& 0.5836& \begin{array}{lll}0.4800& 0.4845& 0.3400\end{array}\\ 0.4164& 0.5& \begin{array}{lll}0.4845& 0.5318& 0.3927\end{array}\\ \begin{array}{c}0.5200\\ 0.5155\\ 0.6600\end{array}& \begin{array}{c}0.5155\\ 0.4682\\ 0.6073\end{array}& \begin{array}{lll}0.5& 0.5736& 0.4764\\ 0.4264& 0.5& 0.3000\\ 0.5236& 0.7000& 0.5\end{array}\end{array}\right),$$

$${B}^{{G}_{2}}=\left(\begin{array}{lll}0.5& 0.5443& \begin{array}{lll}0.3643& 0.3600& 0.4029\end{array}\\ 0.4557& 0.5& \begin{array}{lll}0.2557& 0.6186& 0.7971\end{array}\\ \begin{array}{c}0.6357\\ 0.6400\\ 0.5971\end{array}& \begin{array}{c}0.7443\\ 0.3814\\ 0.2029\end{array}& \begin{array}{lll}0.5& 0.2086& 0.3629\\ 0.7914& 0.5& 0.3114\\ 0.6371& 0.6886& 0.5\end{array}\end{array}\right),$$

$${B}^{{G}_{3}}=\left(\begin{array}{lll}0.5& 0.7450& \begin{array}{lll}0.4800& 0.0700& 0.3550\end{array}\\ 0.2550& 0.5& \begin{array}{lll}0.4050& 0.7650& 0.9950\end{array}\\ \begin{array}{c}0.5200\\ 0.9300\\ 0.6450\end{array}& \begin{array}{c}0.5950\\ 0.2350\\ 0.0050\end{array}& \begin{array}{lll}0.5& 0.8350& 0.5800\\ 0.1650& 0.5& 0.3150\\ 0.4200& 0.6850& 0.5\end{array}\end{array}\right).$$

Based on Eq. (17), the similarity matrices are obtained as follows.

$${\text{SM}}_{12}=\left(\begin{array}{lll}-& 0.9606& \begin{array}{lll}0.8843& 0.8755& 0.9371\end{array}\\ 0.9606& -& \begin{array}{lll}0.7712& 0.9132& 0.5956\end{array}\\ \begin{array}{c}0.8843\\ 0.8755\\ 0.9371\end{array}& \begin{array}{c}0.7712\\ 0.9132\\ 0.5956\end{array}& \begin{array}{lll}-& 0.6349& 0.8865\\ 0.6349& -& 0.9886\\ 0.8865& 0.9886& -\end{array}\end{array}\right),$$

$${\text{SM}}_{13}=\left(\begin{array}{lll}-& 0.8386& \begin{array}{lll}1.0000& 0.5855& 0.9850\end{array}\\ 0.8386& -& \begin{array}{lll}0.9205& 0.7668& 0.3977\end{array}\\ \begin{array}{c}1\\ 0.5855\\ 0.9850\end{array}& \begin{array}{c}0.9205\\ 0.7668\\ 0.3977\end{array}& \begin{array}{lll}-& 0.7386& 0.8964\\ 0.7386& -& 0.9850\\ 0.8964& 0.9850& -\end{array}\end{array}\right),$$

$${\text{SM}}_{23}=\left(\begin{array}{lll}-& 0.7993& \begin{array}{lll}0.8843& 0.7100& 0.9521\end{array}\\ 0.7993& -& \begin{array}{lll}0.8507& 0.8536& 0.8021\end{array}\\ \begin{array}{c}0.8843\\ 0.7100\\ 0.9521\end{array}& \begin{array}{c}0.8507\\ 0.8536\\ 0.8021\end{array}& \begin{array}{lll}-& 0.3736& 0.7829\\ 0.3736& -& 0.9964\\ 0.7829& 0.9964& -\end{array}\end{array}\right).$$

From Eqs. (18)–(20), we can obtain the consensus degrees for each alternative.

$${ca}_{1}=0.8926, {ca}_{2}=0.7967, {ca}_{3}=0.8038, {ca}_{4}=0.8219, {ca}_{5}=0.8488.$$

Therefore, according to Eq. (21) the global consensus level is calculated.

$${cl}_{2}=0.8328.$$

As ${cl}_{2}<0.85$, the feedback adjustment strategy is provided. According to Eq. (22), we conclude the preferences over the alternatives ${x}_{2}$ and ${x}_{3}$ are supposed to be adjusted. According to Eq. (23), we further conclude the preferences over these pairs of alternatives $\left({x}_{2},{x}_{3}\right)$, $\left({x}_{2},{x}_{5}\right)$ and $\left({x}_{3},{x}_{4}\right)$ need to be adjusted. According to Eqs. (24) and (26), the ideal cluster and all the other clusters are presented as follows.

$${\text{CLT}}_{23}^{+}=\left\{{G}_{3}\right\}, {\text{CLT}}_{23}^{- \prime}=\left\{{G}_{1},{G}_{2}\right\},$$

$${\text{CLT}}_{25}^{+}=\left\{{G}_{2}\right\}, {\text{CLT}}_{25}^{- \prime}=\left\{{G}_{1},{G}_{3}\right\},$$

$${\text{CLT}}_{34}^{+}=\left\{{G}_{1}\right\}, {\text{CLT}}_{34}^{- \prime}=\left\{{G}_{2},{G}_{3}\right\}.$$

### Third Round

From Eqs. (29)–(31), the DMs who need to adjust the preferences are identified, and the updated preferences for DMs are listed below.

$${B}^{\mathrm{1,3}}=\left(\begin{array}{lll}0.5& 0.56& \begin{array}{lll}0.36& 0.44& 0.40\end{array}\\ 0.44& 0.5& \begin{array}{lll}0.46& 0.96& 0.86\end{array}\\ \begin{array}{c}0.64\\ 0.56\\ 0.60\end{array}& \begin{array}{c}0.54\\ 0.04\\ 0.14\end{array}& \begin{array}{lll}0.5& 0.52& 0.30\\ 0.48& 0.5& 0.12\\ 0.70& 0.88& 0.5\end{array}\end{array}\right), \quad {B}^{\mathrm{2,3}}=\left(\begin{array}{lll}0.5& 0.35& \begin{array}{lll}0.47& 0.36& 0.38\end{array}\\ 0.65& 0.5& \begin{array}{lll}0.36& 0.61& 0.88\end{array}\\ \begin{array}{c}0.53\\ 0.64\\ 0.62\end{array}& \begin{array}{c}0.64\\ 0.39\\ 0.12\end{array}& \begin{array}{lll}0.5& 0.54& 0.78\\ 0.46& 0.5& 0.50\\ 0.22& 0.50& 0.5\end{array}\end{array}\right),$$

$${B}^{\mathrm{3,3}}=\left(\begin{array}{lll}0.5& 0.22& \begin{array}{lll}0.51& 0.89& 0.33\end{array}\\ 0.78& 0.5& \begin{array}{lll}0.32& 0.96& 0.78\end{array}\\ \begin{array}{c}0.49\\ 0.11\\ 0.67\end{array}& \begin{array}{c}0.68\\ 0.04\\ 0.22\end{array}& \begin{array}{lll}0.5& 0.93& 0.01\\ 0.07& 0.5& 0.28\\ 0.99& 0.72& 0.5\end{array}\end{array}\right), \quad {B}^{\mathrm{4,3}}=\left(\begin{array}{lll}0.5& 0.69& \begin{array}{lll}0.05& 0.32& 0.49\end{array}\\ 0.31& 0.5& \begin{array}{lll}0.50& 0.79& 0.81\end{array}\\ \begin{array}{c}0.95\\ 0.68\\ 0.51\end{array}& \begin{array}{c}0.50\\ 0.21\\ 0.19\end{array}& \begin{array}{lll}0.5& 0.51& 0.49\\ 0.49& 0.5& 0.33\\ 0.51& 0.67& 0.5\end{array}\end{array}\right),$$

$${B}^{\mathrm{5,3}}=\left(\begin{array}{lll}0.5& 0.87& \begin{array}{lll}0.83& 0.49& 0.58\end{array}\\ 0.13& 0.5& \begin{array}{lll}0.33& 0.98& 0.88\end{array}\\ \begin{array}{c}0.17\\ 0.51\\ 0.42\end{array}& \begin{array}{c}0.67\\ 0.02\\ 0.12\end{array}& \begin{array}{lll}0.5& 0.51& 0.14\\ 0.49& 0.5& 0.36\\ 0.86& 0.64& 0.5\end{array}\end{array}\right), \quad {B}^{\mathrm{6,3}}=\left(\begin{array}{lll}0.5& 0.43& \begin{array}{lll}0.40& 0.31& 0.42\end{array}\\ 0.57& 0.5& \begin{array}{lll}0.40& 0.92& 0.75\end{array}\\ \begin{array}{c}0.60\\ 0.69\\ 0.58\end{array}& \begin{array}{c}0.60\\ 0.08\\ 0.25\end{array}& \begin{array}{lll}0.5& 0.54& 0.26\\ 0.46& 0.5& 0.41\\ 0.74& 0.59& 0.5\end{array}\end{array}\right)$$,

$${B}^{\mathrm{7,3}}=\left(\begin{array}{lll}0.5& 0.22& \begin{array}{lll}0.41& 0.47& 0.40\end{array}\\ 0.78& 0.5& \begin{array}{lll}0.48& 0.09& 0.76\end{array}\\ \begin{array}{c}0.59\\ 0.53\\ 0.60\end{array}& \begin{array}{c}0.52\\ 0.91\\ 0.24\end{array}& \begin{array}{lll}0.5& 0.48& 0.37\\ 0.52& 0.5& 0.18\\ 0.63& 0.82& 0.5\end{array}\end{array}\right), \quad {B}^{\mathrm{8,3}}=\left(\begin{array}{lll}0.5& 0.62& \begin{array}{lll}0.19& 0.46& 0.12\end{array}\\ 0.38& 0.5& \begin{array}{lll}0.43& 0.75& 0.74\end{array}\\ \begin{array}{c}0.81\\ 0.54\\ 0.88\end{array}& \begin{array}{c}0.57\\ 0.25\\ 0.26\end{array}& \begin{array}{lll}0.5& 0.25& 0.02\\ 0.75& 0.5& 0.33\\ 0.98& 0.67& 0.50\end{array}\end{array}\right),$$

$${B}^{\mathrm{9,3}}=\left(\begin{array}{lll}0.5& 0.27& \begin{array}{lll}0.48& 0.33& 0.42\end{array}\\ 0.73& 0.5& \begin{array}{lll}0.37& 0.32& 0.75\end{array}\\ \begin{array}{c}0.52\\ 0.67\\ 0.58\end{array}& \begin{array}{c}0.63\\ 0.68\\ 0.25\end{array}& \begin{array}{lll}0.5& 0.39& 0.68\\ 0.61& 0.5& 0.38\\ 0.32& 0.62& 0.5\end{array}\end{array}\right), \quad {B}^{\mathrm{10,3}}=\left(\begin{array}{lll}0.5& 0.94& \begin{array}{lll}0.25& 0.47& 0.19\end{array}\\ 0.06& 0.5& \begin{array}{lll}0.38& 0.28& 0.77\end{array}\\ \begin{array}{c}0.75\\ 0.53\\ 0.81\end{array}& \begin{array}{c}0.62\\ 0.72\\ 0.23\end{array}& \begin{array}{lll}0.5& 0.93& 0.88\\ 0.07& 0.5& 0.36\\ 0.12& 0.64& 0.5\end{array}\end{array}\right),$$

$${B}^{\mathrm{11,3}}=\left(\begin{array}{lll}0.5& 0.71& \begin{array}{lll}0.49& 0.41& 0.40\end{array}\\ 0.29& 0.5& \begin{array}{lll}0.32& 0.38& 0.72\end{array}\\ \begin{array}{c}0.51\\ 0.59\\ 0.60\end{array}& \begin{array}{c}0.68\\ 0.62\\ 0.28\end{array}& \begin{array}{lll}0.5& 0.53& 0.39\\ 0.47& 0.5& 0.56\\ 0.61& 0.44& 0.5\end{array}\end{array}\right), \quad {B}^{\mathrm{12,3}}=\left(\begin{array}{lll}0.5& 0.95& \begin{array}{lll}0.26& 0.44& 0.05\end{array}\\ 0.05& 0.5& \begin{array}{lll}0.49& 0.76& 0.74\end{array}\\ \begin{array}{c}0.74\\ 0.56\\ 0.95\end{array}& \begin{array}{c}0.51\\ 0.24\\ 0.26\end{array}& \begin{array}{lll}0.5& 0.25& 0.89\\ 0.75& 0.5& 0.31\\ 0.11& 0.69& 0.5\end{array}\end{array}\right),$$

$${B}^{\mathrm{13,3}}=\left(\begin{array}{lll}0.5& 0.69& \begin{array}{lll}0.46& 0.12& 0.30\end{array}\\ 0.31& 0.5& \begin{array}{lll}0.71& 0.72& 0.86\end{array}\\ \begin{array}{c}0.54\\ 0.88\\ 0.70\end{array}& \begin{array}{c}0.29\\ 0.28\\ 0.14\end{array}& \begin{array}{lll}0.5& 0.61& 0.76\\ 0.39& 0.5& 0.38\\ 0.24& 0.62& 0.5\end{array}\end{array}\right), \quad {B}^{\mathrm{14,3}}=\left(\begin{array}{lll}0.5& 0.67& \begin{array}{lll}0.35& 0.36& 0.42\end{array}\\ 0.33& 0.5& \begin{array}{lll}0.50& 0.42& 0.71\end{array}\\ \begin{array}{c}0.65\\ 0.64\\ 0.58\end{array}& \begin{array}{c}0.50\\ 0.58\\ 0.29\end{array}& \begin{array}{lll}0.5& 0.69& 0.94\\ 0.31& 0.5& 0.11\\ 0.06& 0.89& 0.5\end{array}\end{array}\right),$$

$${B}^{\mathrm{15,3}}=\left(\begin{array}{lll}0.5& 0.80& \begin{array}{lll}0.50& 0.02& 0.41\end{array}\\ 0.20& 0.5& \begin{array}{lll}0.10& 0.81& 0.80\end{array}\\ \begin{array}{c}0.50\\ 0.98\\ 0.59\end{array}& \begin{array}{c}0.90\\ 0.19\\ 0.20\end{array}& \begin{array}{lll}0.5& 0.62& 0.40\\ 0.38& 0.5& 0.25\\ 0.60& 0.75& 0.5\end{array}\end{array}\right), \quad {B}^{\mathrm{16,3}}=\left(\begin{array}{lll}0.5& 0.70& \begin{array}{lll}0.36& 0.30& 0.29\end{array}\\ 0.30& 0.5& \begin{array}{lll}0.34& 0.01& 0.84\end{array}\\ \begin{array}{c}0.64\\ 0.70\\ 0.71\end{array}& \begin{array}{c}0.66\\ 0.99\\ 0.16\end{array}& \begin{array}{lll}0.5& 0.52& 0.19\\ 0.48& 0.5& 0.22\\ 0.81& 0.78& 0.5\end{array}\end{array}\right),$$

$${B}^{\mathrm{17,3}}=\left(\begin{array}{lll}0.5& 0.37& \begin{array}{lll}0.42& 0.38& 0.44\end{array}\\ 0.63& 0.5& \begin{array}{lll}0.33& 0.66& 0.72\end{array}\\ \begin{array}{c}0.58\\ 0.62\\ 0.56\end{array}& \begin{array}{c}0.67\\ 0.34\\ 0.28\end{array}& \begin{array}{lll}0.5& 0.48& 0.13\\ 0.52& 0.5& 0.04\\ 0.87& 0.96& 0.5\end{array}\end{array}\right), \quad {B}^{\mathrm{18,3}}=\left(\begin{array}{lll}0.5& 0.55& \begin{array}{lll}0.52& 0.53& 0.26\end{array}\\ 0.45& 0.5& \begin{array}{lll}0.50& 0.17& 0.75\end{array}\\ \begin{array}{c}0.48\\ 0.47\\ 0.74\end{array}& \begin{array}{c}0.50\\ 0.83\\ 0.25\end{array}& \begin{array}{lll}0.5& 0.26& 0.26\\ 0.74& 0.5& 0.27\\ 0.74& 0.73& 0.5\end{array}\end{array}\right),$$

$${B}^{\mathrm{19,3}}=\left(\begin{array}{lll}0.5& 0.68& \begin{array}{lll}0.89& 0.49& 0.67\end{array}\\ 0.32& 0.5& \begin{array}{lll}0.47& 0.48& 0.79\end{array}\\ \begin{array}{c}0.11\\ 0.51\\ 0.33\end{array}& \begin{array}{c}0.53\\ 0.52\\ 0.21\end{array}& \begin{array}{lll}0.5& 0.67& 0.43\\ 0.33& 0.5& 0.36\\ 0.57& 0.64& 0.5\end{array}\end{array}\right), \quad {B}^{\mathrm{20,3}}=\left(\begin{array}{lll}0.5& 0.43& \begin{array}{lll}0.59& 0.40& 0.30\end{array}\\ 0.57& 0.5& \begin{array}{lll}0.47& 0.64& 0.77\end{array}\\ \begin{array}{c}0.41\\ 0.50\\ 0.70\end{array}& \begin{array}{c}0.53\\ 0.36\\ 0.23\end{array}& \begin{array}{lll}0.5& 0.95& 0.62\\ 0.05& 0.5& 0.36\\ 0.38& 0.64& 0.5\end{array}\end{array}\right)$$.

Based on the historical preference relations, use the *k*-means clustering technique to classify DMs into the following clusters.

$${G}_{1}=\left\{{B}^{\mathrm{10,3}},{B}^{\mathrm{12,3}},{B}^{\mathrm{13,3}},{B}^{\mathrm{14,3}},{B}^{\mathrm{20,3}}\right\},$$

$${G}_{2}=\left\{{B}^{\mathrm{1,3}},{B}^{\mathrm{2,3}},{B}^{\mathrm{3,3}},{B}^{\mathrm{4,3}},{B}^{\mathrm{5,3}},{B}^{\mathrm{6,3}},{B}^{\mathrm{8,3}},{B}^{\mathrm{11,3}},{B}^{\mathrm{15,3}},{B}^{\mathrm{17,3}},{B}^{\mathrm{19,3}}\right\},$$

$${G}_{3}=\left\{{B}^{\mathrm{7,3}},{B}^{\mathrm{9,3}},{B}^{\mathrm{16,3}},{B}^{\mathrm{18,3}}\right\}.$$

Based on Eq. (15), we can obtain the collective preferences for each cluster.

$${B}^{{G}_{1}}=\left(\begin{array}{lll}0.5& 0.7360& \begin{array}{lll}0.3820& 0.3580& 0.2520\end{array}\\ 0.2640& 0.5& \begin{array}{lll}0.5100& 0.5640& 0.7700\end{array}\\ \begin{array}{c}0.6180\\ 0.6420\\ 0.7480\end{array}& \begin{array}{c}0.4900\\ 0.4360\\ 0.2300\end{array}& \begin{array}{lll}0.5& 0.6860& 0.8180\\ 0.3140& 0.5& 0.3040\\ 0.1820& 0.6960& 0.5\end{array}\end{array}\right),$$

$${B}^{{G}_{2}}=\left(\begin{array}{lll}0.5& 0.5727& \begin{array}{lll}0.4645& 0.4155& 0.4218\end{array}\\ 0.4273& 0.5& \begin{array}{lll}0.3655& 0.7545& 0.7936\end{array}\\ \begin{array}{c}0.5355\\ 0.5845\\ 0.5782\end{array}& \begin{array}{c}0.6345\\ 0.2455\\ 0.2064\end{array}& \begin{array}{lll}0.5& 0.5545& 0.3045\\ 0.4455& 0.5& 0.3218\\ 0.6955& 0.6782& 0.5\end{array}\end{array}\right),$$

$${B}^{{G}_{3}}=\left(\begin{array}{lll}0.5& 0.4350& \begin{array}{lll}0.4425& 0.4075& 0.3425\end{array}\\ 0.5650& 0.5& \begin{array}{lll}0.4225& 0.1475& 0.7750\end{array}\\ \begin{array}{c}0.5575\\ 0.5925\\ 0.6575\end{array}& \begin{array}{c}0.5775\\ 0.8525\\ 0.2250\end{array}& \begin{array}{lll}0.5& 0.4125& 0.3750\\ 0.5875& 0.5& 0.2625\\ 0.6250& 0.7375& 0.5\end{array}\end{array}\right)$$.

Based on Eq. (17), the similarity matrices are obtained as follows.

$${\text{SM}}_{12}=\left(\begin{array}{lll}-& 0.8367& \begin{array}{lll}0.9175& 0.9425& 0.8302\end{array}\\ 0.8367& -& \begin{array}{lll}0.8555& 0.8095& 0.9764\end{array}\\ \begin{array}{c}0.9175\\ 0.9425\\ 0.8302\end{array}& \begin{array}{c}0.8555\\ 0.8095\\ 0.9764\end{array}& \begin{array}{lll}-& 0.8685& 0.4865\\ 0.8685& -& 0.9822\\ 0.4865& 0.9822& -\end{array}\end{array}\right),$$

$${\text{SM}}_{13}=\left(\begin{array}{lll}-& 0.6990& \begin{array}{lll}0.9395& 0.9505& 0.9095\end{array}\\ 0.6990& -& \begin{array}{lll}0.9125& 0.5835& 0.9950\end{array}\\ \begin{array}{c}0.9395\\ 0.9505\\ 0.9095\end{array}& \begin{array}{c}0.9125\\ 0.5835\\ 0.9950\end{array}& \begin{array}{lll}-& 0.7265& 0.5570\\ 0.7265& -& 0.9585\\ 0.5570& 0.9585& -\end{array}\end{array}\right),$$

$${\text{SM}}_{23}=\left(\begin{array}{lll}-& 0.8623& \begin{array}{lll}0.9780& 0.9920& 0.9207\end{array}\\ 0.8623& -& \begin{array}{lll}0.9430& 0.3930& 0.9814\end{array}\\ \begin{array}{c}0.9780\\ 0.9920\\ 0.9207\end{array}& \begin{array}{c}0.9430\\ 0.3930\\ 0.9814\end{array}& \begin{array}{lll}-& 0.8580& 0.9295\\ 0.8580& -& 0.9407\\ 0.9295& 0.9407& -\end{array}\end{array}\right).$$

From Eqs. (18)–(20), we can obtain the consensus degrees for each alternative.

$${ca}_{1}=0.9014, {ca}_{2}=0.8298, {ca}_{3}=0.8360, {ca}_{4}=0.8458, {ca}_{5}=0.8708.$$

Therefore, according to Eq. (21) the global consensus level is calculated.

$${cl}_{3}=0.8568.$$

Since ${cl}_{3}>0.85$, it is concluded that the consensus threshold is reached. We can further obtain the collective preference relation according to Eq. (2).

$${P}^{c}=\left(\begin{array}{lll}0.5& 0.5860& \begin{array}{lll}0.4395& 0.3995& 0.3635\end{array}\\ 0.4140& 0.5& \begin{array}{lll}0.4130& 0.5855& 0.7840\end{array}\\ \begin{array}{c}0.5605\\ 0.6005\\ 0.6365\end{array}& \begin{array}{c}0.5870\\ 0.4145\\ 0.2160\end{array}& \begin{array}{lll}0.5& 0.5590& 0.4470\\ 0.4410& 0.5& 0.3055\\ 0.5530& 0.6945& 0.5\end{array}\end{array}\right).$$

Then, based on Eq. (3) the collective ranking of alternatives is ${x}_{2}\succ {x}_{3}\succ {x}_{5}\succ {x}_{1}\succ {x}_{4}$.

## Simulation and Comparison Analysis

In this section, the simulation experiments and comparison analysis are designed to show the validity of the CMHP-LCRP framework.

### Simulation Experiment

In the simulation experiment, at first the initial preference relations of DMs are randomly generated and further transformed into the historical preference format. Based on these historical preference data, we use the k-means clustering technique to classify DMs into several clusters. Further, the consensus level is computed. If the consensus level fails to reach the consensus threshold, then it comes to the local feedback adjustment stage. According to Eqs. (22), (23), (26) and (28), the alternatives, the pairs of alternatives, the clusters and the DMs where the preferences need to be adjusted are identified. Next, the identified DMs are supposed to adjust their preference over the identified positions based on Eqs. (29)–(31) and we obtain the updated preference relations of DMs. In the next round, the updated preference relations, together with all the previous preference relations, are transformed into the historical preference format again and it comes to the next clustering process. All the processes loop until the pre-defined consensus threshold is reached or it comes to the maximum number of rounds allowed.

The detailed simulation method for the CMHP-LCRP framework (${\text{SM}}$) is given below.

**Input**: $$m,n, Q, \beta ,\overline{cl }$$ and $${z}_{max}$$.

**Output**: $$z$$ and $$s$$.

**Step 1**: Let $z=1$, initialize DMs' preference relations and weights. We randomly generate $m$ $n\times n$ preference relations ${B}^{k}={\left[{b}_{ij}^{k}\right]}_{n\times n}\left(k=\mathrm{1,2},\dots ,m\right)$, and ${B}^{k,z}={B}^{k}$. The weights of DMs are supposed to be the same, i.e., ${w}_{k}=1/m(k=\mathrm{1,2},\dots ,m)$.

**Step 2**: Gather the individuals' preference relations in each round $\left\{{B}^{k,z},{B}^{k,z-1},\dots ,{B}^{k,1}\right\}\left(k=\mathrm{1,2},\dots ,m\right)$ and transform them into the historical preference relations ${H}^{k,z}\left(k=\mathrm{1,2},\dots ,m\right)$ according to Eqs. (8) and (9).

**Step 3**: Use the *k*-means clustering technique to classify DMs into $Q$ clusters $\left\{{G}_{1},{G}_{2},\dots ,{G}_{Q}\right\}$ based on ${H}^{k,z}\left(k=\mathrm{1,2},\dots ,m\right)$.

**Step 4:** Compute the global consensus level among DMs ${cl}_{z}$ according to Eq. (21). If ${cl}_{z}\ge \overline{cl }$ or $z\ge {z}_{\mathrm{max}}$, go to step 7; otherwise continue with the next step.

**Step 5:** According to the identification rules described in Sect. 4.2.2, the alternatives, the pairs of alternatives, the clusters and the DMs where the preferences need to be adjusted are identified. The identified set of DMs over the identified positions is denoted as ${E}_{ij}^{-}$.

**Step 6:** Feedback adjustment. For the other DMs except those in ${E}_{ij}^{-}$, the preferences over the pair of alternatives $\left({x}_{i},{x}_{j}\right)$ keep unchanged in the next round. For the DM ${e}_{k}$ in ${E}_{ij}^{-}$, use the following rule to modify the preferences.

$$\left\{\begin{array}{l}{b}_{ij}^{k,z+1}=\tau {b}_{ij}^{{G}_{{s}^{+}},z}+(1-\tau ){b}_{ij}^{k,z} \\ {b}_{ij}^{k,z+1}={b}_{ij}^{{G}_{{s}^{+}},z}+\beta \varphi \end{array}\right. ,$$

where the value of $\tau$ is uniformly randomly generated from the interval $\left[0, 1\right]$ and it guarantees ${b}_{ij}^{k,z+1}\in [min({b}_{ij}^{k,z},{b}_{ij}^{{G}_{{s}^{+}},z}),max({b}_{ij}^{k,z},{b}_{ij}^{{G}_{{s}^{+}},z})]$. The value of $\varphi$ is uniformly randomly generated from the interval $\left[-1, 1\right]$, and it guarantees the preference difference between ${b}_{ij}^{k,z+1}$ and ${b}_{ij}^{{G}_{{s}^{+}},z}$ is no more than the threshold $\beta$. Let $z=z+1$, and go to step 2.

**Step 7:** Output. If ${cl}_{z}\ge \overline{cl }$, $s=1$; otherwise, $s=0$. Output $z$ and $s$.

**Note.** In the simulation method, (1) $m$, $n$ and $Q$ represent the number of DMs, alternatives and clusters respectively; $\overline{cl }$ and ${z}_{max}$ represent the consensus threshold and the maximum number of rounds allowed respectively. (2) The parameter $\beta$ denotes the coefficient to guide direction for preference adjustment. The smaller the value of $\beta$, the larger the degree to adjust the preferences of DMs. (3) The output parameter $z$ denotes the iteration number to reach the consensus threshold. The smaller the value of $z$, the faster the speed to reach the consensus threshold. (4) The parameter $s$ reflects whether the consensus can be achieved or not. If the consensus is achieved within the maximum number of rounds allowed, $s=1$; otherwise, $s=0$.

Let ${z}_{max}=5$ and $\overline{cl }=0.85$, and different input parameters $m, \; n$, $Q, \; \beta$ are set for the simulation experiment. To be specific, we run the simulations method ($\mathrm{SM}$) 1000 times to obtain ${\text{AZ}}$ and ${\text{AS}}$, which represent the average values of $z$ and $s$ respectively. The indicator ${\text{AZ}}$ reflects the average iteration number to reach the consensus threshold, and that of ${\text{AS}}$ reflects the success ratio of achieving a consensus. Under different input parameters, the results are listed in Table 1.

**Table 1:** ${\text{AZ}}$ and ${\text{AS}}$ under different parameters

		$Q=3$						$Q=4$						$Q=5$					
		$\beta =0.1$		$\beta =0.2$		$\beta =0.3$		$\beta =0.1$		$\beta =0.2$		$\beta =0.3$		$\beta =0.1$		$\beta =0.2$		$\beta =0.3$	
$m$	$n$	$\mathrm{AZ}$	$\mathrm{AS}$	$\mathrm{AZ}$	$\mathrm{AS}$	$\mathrm{AZ}$	$\mathrm{AS}$	$\mathrm{AZ}$	$\mathrm{AS}$	$\mathrm{AZ}$	$\mathrm{AS}$	$\mathrm{AZ}$	$\mathrm{AS}$	$\mathrm{AZ}$	$\mathrm{AS}$	$\mathrm{AZ}$	$\mathrm{AS}$	$\mathrm{AZ}$	$\mathrm{AS}$
20	4	2.402	0.976	2.810	0.951	3.823	0.699	2.427	0.967	2.774	0.955	3.823	0.696	2.384	0.964	2.846	0.945	4.209	0.489
	5	2.293	0.995	2.565	0.983	3.333	0.857	2.209	0.995	2.544	0.985	3.432	0.854	2.204	0.996	2.598	0.985	3.944	0.683
	6	2.021	1.000	2.348	0.996	3.064	0.957	2.071	0.998	2.338	0.998	3.023	0.961	2.077	1.000	2.448	0.998	3.607	0.831
30	4	2.352	0.976	2.723	0.939	3.566	0.807	2.352	0.966	2.630	0.970	3.563	0.804	2.311	0.973	2.686	0.951	4.050	0.617
	5	2.004	0.999	2.248	0.994	2.813	0.955	2.069	0.998	2.341	0.998	3.029	0.953	2.090	0.997	2.446	0.992	3.408	0.881
	6	1.753	1.000	1.969	1.000	2.519	0.995	1.756	1.000	1.974	1.000	2.474	0.998	1.894	1.000	2.143	1.000	2.865	0.978
40	4	2.276	0.973	2.610	0.962	3.444	0.835	2.323	0.970	2.617	0.963	3.419	0.842	2.236	0.976	2.626	0.951	3.777	0.724
	5	1.901	0.998	2.060	0.997	2.549	0.976	1.934	0.999	2.175	0.998	2.771	0.975	2.002	0.998	2.296	0.996	3.062	0.947
	6	1.544	1.000	1.745	1.000	2.089	1.000	1.578	1.000	1.749	1.000	2.037	1.000	1.705	1.000	1.940	1.000	2.441	0.997
50	4	2.410	0.978	2.658	0.965	3.349	0.870	2.299	0.976	2.582	0.963	3.291	0.868	2.305	0.966	2.634	0.943	3.646	0.769
	5	1.741	1.000	1.919	1.000	2.325	0.992	1.831	1.000	2.113	0.999	2.586	0.991	1.977	1.000	2.196	0.997	2.869	0.967
	6	1.431	1.000	1.563	1.000	1.840	1.000	1.431	1.000	1.563	1.000	1.840	1.000	1.609	1.000	1.783	1.000		

From Table 1, the following observations can be obtained.

1. In general, it takes an average of 2–3 rounds to reach the consensus threshold, and it has high success ratios of achieving a consensus (close to 1) for most cases in the CMHP-LCRP framework.
2. With increasing $m$ and $n$ values, the value ${\text{AZ}}$ decreases and that of ${\text{AS}}$ increases. The finding implies that more DMs or alternatives involved will increase the speed to reach the consensus threshold and will improve the success ratio of achieving a consensus in the CMHP-LCRP framework. With increasing $Q$ values, on the whole the value ${\text{AZ}}$ shows increasing trend while that of ${\text{AS}}$ shows decreasing trend. The finding implies that a relatively small number of clusters may increase the speed to reach the consensus threshold and shows higher success ratio of achieving a consensus.
3. With increasing $\beta$ values, the value ${\text{AZ}}$ increases and that of ${\text{AS}}$ decreases. In the CMHP-LCRP framework the smaller the value of $\beta$, the more the DMs that are supposed to adjust their preferences. Therefore, it is faster to reach the consensus threshold, and the success ratio of achieving a consensus is higher by comparison.

### Comparison Analysis

As for LSGDM problem, clustering is a widely used tool. In this paper we propose a CMHP-LCRP framework in which all the historical preferences of DMs are employed for clustering. However, the traditional LCRP models are based on a clustering method using just the latest round of preference information of DMs, which may fail to fully reflect the change of DMs' preferences. In order to show the validity of the CMHP-LCRP framework, we aim to compare the CMHP-LCRP framework with the traditional LCRP framework.

The simulation method for the traditional LCRP framework (${\text{SM}}^{\prime}$) is given below. Considering the Input, Output, Step 1 and Steps 3–7 are the same as ${\text{SM}}$ for the CMHP-LCRP framework, we omit them. We just need to replace Step 2 with Step 2' for ${\text{SM}}^{\prime}$.

**Step 2'**: Transform ${B}^{k,z}\left(k=\mathrm{1,2},\dots ,m\right)$ into a vector that consists of its upper triangular elements based on Eqs. (6) and (7).

Considering that input parameters may have influence on the simulation results, three groups of comparisons are conducted by using different input parameters: (1) different input parameters $m$ and $n$; (2) different input parameters $m$ and $Q$; (3) different input parameters $m$ and $\beta$.

**Fig. 2:**
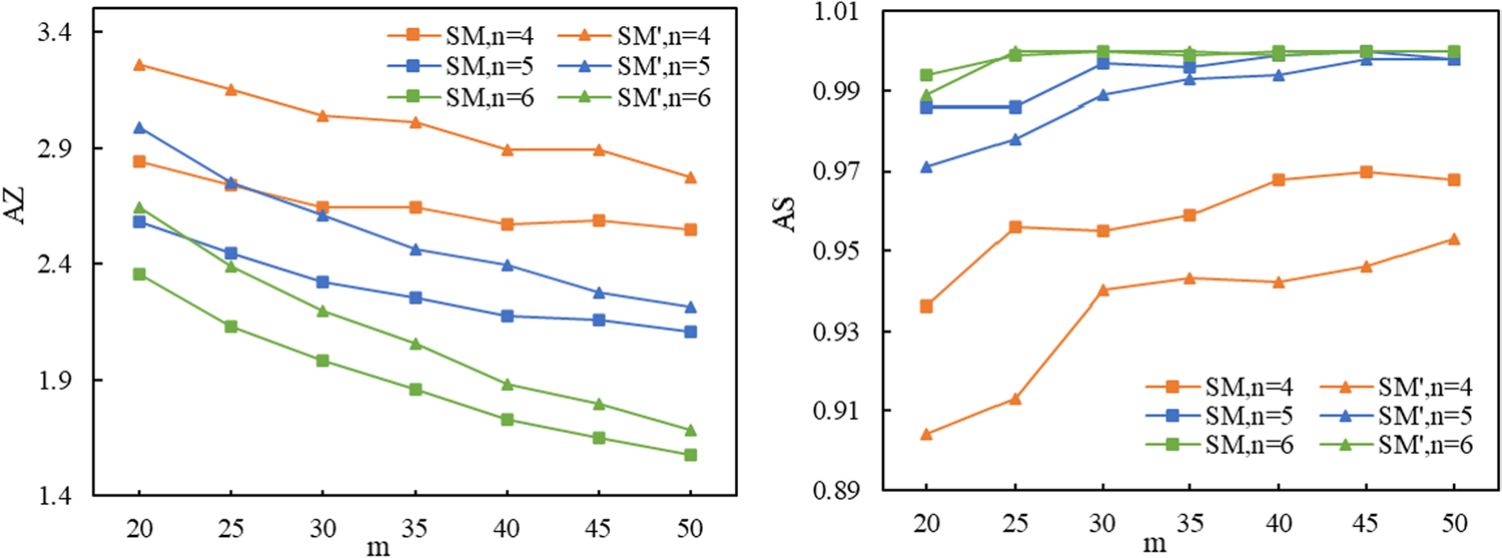
${\text{AZ}}$ and ${\text{AS}}$ under different parameters $m$ and $n$

For the first comparison analysis, let ${z}_{max}=5$, $\overline{cl }=0.85$, $Q=4$ and $\beta =0.2$, different input parameters $m$ and $n$ are set and we run the simulations methods ${\text{SM}}$ and ${\text{SM}}^{\prime}$ 1000 times to obtain values ${\text{AZ}}$ and ${\text{AS}}$ using the k-means clustering technique. The results are described in Fig. 2.

**Fig. 3:**
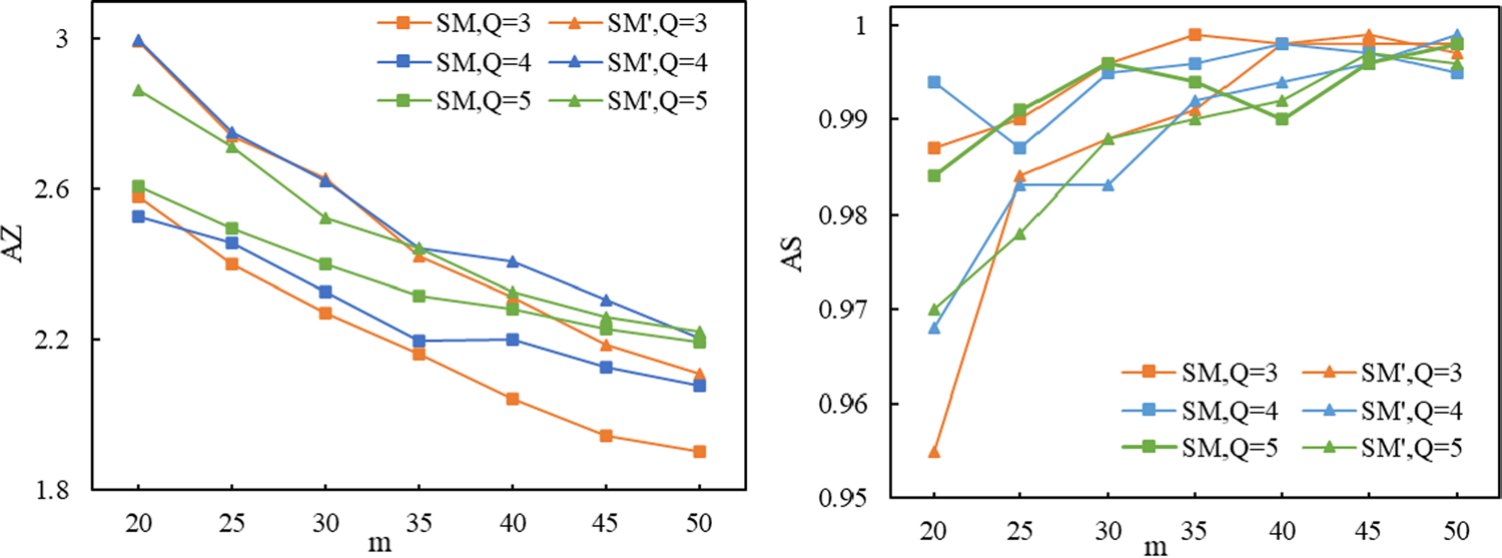
${\text{AZ}}$ and ${\text{AS}}$ under different parameters $m$ and $Q$

For the second comparison analysis, let ${z}_{max}=5$, $\overline{cl }=0.85$, $n=5$ and $\beta =0.2$, different input parameters $m$ and $Q$ are set and we run the simulations methods ${\text{SM}}$ and ${\text{SM}}^{\prime}$ 1000 times to obtain values ${\text{AZ}}$ and ${\text{AS}}$ using the k-means clustering technique. The results are described in Fig. 3.

**Fig. 4:**
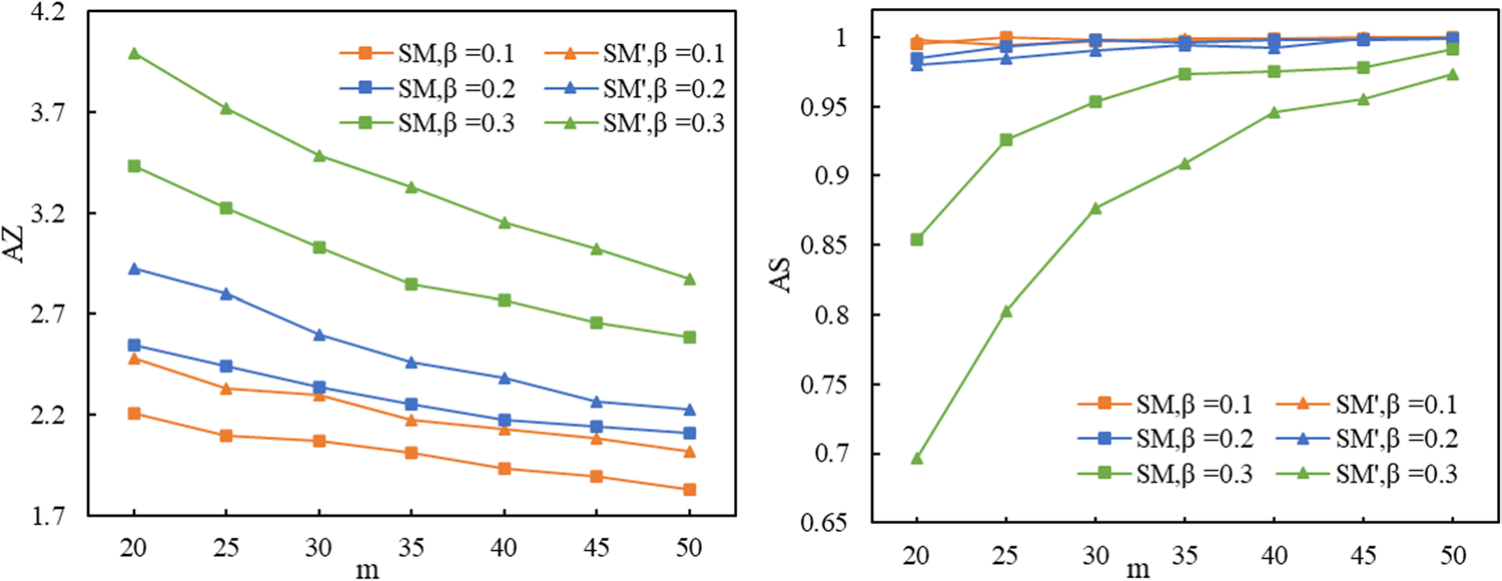
${\text{AZ}}$ and ${\text{AS}}$ under different parameters $m$ and $\beta$

For the third comparison analysis, let ${z}_{max}=5$, $\overline{cl }=0.85$, $n=5$ and $Q=4$, different input parameters $m$ and $\beta$ are set and we run the simulations methods ${\text{SM}}$ and ${\text{SM}}^{\prime}$ 1000 times to obtain values ${\text{AZ}}$ and ${\text{AS}}$ using the k-means clustering technique. The results are described in Fig. 4.

From Figs. 2, 3 and 4, the following observations can be obtained.

1. No matter how the input parameters change, the values ${\text{AZ}}$ in the proposed CMHP-LCRP framework are smaller than those in the traditional LCRP framework. It indicates the proposed CMHP-LCRP framework can increase the speed to reach the consensus threshold. Meanwhile, the values ${\text{AS}}$ in the proposed CMHP-LCRP framework are larger than those in the traditional LCRP framework. It indicates the success ratio of achieving a consensus is higher in the CMHP-LCRP framework by comparison.
2. When setting different input parameters, the values of ${\text{AZ}}$ and ${\text{AS}}$ are different. As for the preference adjustment parameter $\beta$, the smaller the value of $\beta$, the more the DMs that need to adjust their preferences and the DMs' updated preferences are closer to each other. Therefore, it is faster to reach the consensus threshold and the success ratio of achieving a consensus is higher. With increasing $m$ and $n$ values, the more the DMs and alternatives involved, so the speed to reach the consensus threshold is faster and the success ratio of achieving a consensus is higher. As for the parameter $Q$, on the whole a relatively small number of clusters may increase the speed to reach the consensus threshold and improve the success ratio of achieving a consensus. It may lie in that fewer clusters result in more stable clustering results and the direction in which DMs adjust the preference is clearer, and it may result in smoother LCPR.

## Conclusion

Clustering is a widely used tool to deal with LSGDM problems. In traditional LCRP framework, just the DMs' preference information in the latest decision round is used for clustering. This paper explores a consensus model using a novel clustering method that takes the historical preference information of DMs in all decision rounds into consideration. By comparison, the historical data can more fully reflect the change of DMs' preferences, thus better guiding the clustering process. To show the validity of the proposed CMHP-LCRP framework, we further compare the CMHP-LCRP framework with the traditional LCRP framework under different input parameters. Compared with the traditional LCRP framework, it is faster to reach the consensus threshold and the success ratio of achieving a consensus is higher for our CMHP-LCRP framework no matter how the input parameters change. The result shows that the CMHP-LCRP framework outperforms the traditional LCRP framework.

Meanwhile, three research directions are interesting for future studies. (1) In this paper, we apply the additive preference relations for analysis. However, there are some other preference representation structures, such as multiplicative preference relations and linguistic preference relations. It is of significance to explore other preference representation structures with historical data to further support the proposed model in this paper. (2) Some DMs may adopt non-cooperative behaviors in the LCRP to achieve their own goal or interests. For instance, some DMs may provide dishonest opinions or refuse to change their evaluations for the sake of their own interests [28, 29]. Therefore, it will be very interesting to explore a more flexible consensus model by taking the non-cooperative behaviors into consideration and build the corresponding mechanism to detect and manage non-cooperative behaviors in the CMHP-LCRP framework in future. (3) The social network among DMs plays an important role in the aggregation process of opinions and the social network analysis has become a hot topic in GDM research [3, 29, 32]. Therefore, it will be interesting to extend the proposed model with the consideration of the social network to provide decision support for practical LCRP problems.

## Data Availability

The datasets generated during the current study are available from the corresponding author on reasonable request.

## Abbreviations

LSGDM: Large-scale group decision-making
LCRP: Large-scale consensus-reaching process
DMs: Decision makers
GDM: Group decision-making
CRP: Consensus-reaching process
CMHP: Clustering method using the historical preferences data
CMHP-LCRP: Large-scale consensus-reaching process framework based on the clustering method using the historical preferences data of decision makers
WA: Weighted average
SM: Simulation method
